# Uncovering novel polyhydroxyalkanoate biosynthesis genes and unique pathway in yeast hanseniaspora valbyensis for sustainable bioplastic production

**DOI:** 10.1038/s41598-024-77382-x

**Published:** 2024-11-08

**Authors:** Desouky A.M. Abd-El-Haleem, Marwa R. Elkatory, Gadallah M. Abu-Elreesh

**Affiliations:** 1 Environmental Biotechnology Department, Genetic Engineering and Biotechnology Institute, City of Scientific Research and Technological Applications SRTA-City, Alexandria, 21934 New Burelarab Egypt; 2 Advanced Technology and New Materials Research Institute, City of Scientific Research and Technological Applications SRTA-City, New Borg El-Arab City, 21934 Alexandria Egypt

**Keywords:** Polyhydroxyalkanoates (PHAs), Budding yeast, *Hanseniaspora valbyensis*, Biosynthesis genes, Biotechnology, Biomaterials, Environmental biotechnology, Metabolic engineering

## Abstract

This study delves into the exploration of polyhydroxyalkanoate (PHA) biosynthesis genes within wild-type yeast strains, spotlighting the exceptional capabilities of isolate DMG-2. Through meticulous screening, DMG-2 emerged as a standout candidate, showcasing vivid red fluorescence indicative of prolific intracellular PHA granules. Characterization via FTIR spectroscopy unveiled a diverse biopolymer composition within DMG-2, featuring distinct functional groups associated with PHA and polyphosphate. Phylogenetic analysis placed DMG-2 within the *Hanseniaspora valbyensis* NRRL Y-1626 group, highlighting its distinct taxonomic classification. Subsequent investigation into DMG-2’s PHA biosynthesis genes yielded promising outcomes, with successful cloning and efficient PHA accumulation confirmed in transgenic *E. coli* cells. Protein analysis of ORF1 revealed its involvement in sugar metabolism, supported by its cellular localization and identification of functional motifs. Genomic analysis revealed regulatory elements within ORF1, shedding light on potential splice junctions and transcriptional networks influencing PHA synthesis pathways. Spectroscopic analysis of the biopolymer extracted from transgenic *E. coli* DMG2-1 provided insights into its co-polymer nature, comprising segments of PHB, PHV, and polyphosphate. GC-MS analysis further elucidated the intricate molecular architecture of the polymer. In conclusion, this study represents a pioneering endeavor in exploring PHA biosynthesis genes within yeast cells, with isolate DMG-2 demonstrating remarkable potential. The findings offer valuable insights for advancing sustainable bioplastic production and hold significant implications for biotechnological applications.

## Introduction

The growing environmental concerns surrounding plastic pollution have galvanized scientific efforts toward the development of sustainable and biodegradable alternatives. Among various biopolymers, polyhydroxyalkanoates (PHAs) have emerged as promising candidates due to their biodegradability, biocompatibility, and versatile material properties^1^. PHAs are polyesters synthesized by numerous microorganisms as intracellular carbon and energy storage compounds^2^. These biopolymers can potentially replace conventional petrochemical plastics in numerous applications, ranging from packaging materials to medical devices^3^. However, the high production costs and limited yields of PHAs have hindered their large-scale commercial adoption, preventing them from completely replacing oil-based polymers^4^.

Therefore, identifying and optimizing microbial strains capable of efficient PHA production is critical for advancing sustainable bioplastic technologies. In this context, yeast isolates represent a compelling avenue for exploration due to their robust growth characteristics, ease of genetic manipulation, and ability to thrive on diverse substrates. Yeasts can utilize inexpensive substrates as carbon sources and can be easily genetically engineered, offering significant advantages for PHA production^5^.

For example, Abd-El-Haleem^6^ screened forty yeast isolates from different Egyptian ecosystems for their ability to produce PHAs. The isolate Rhodotorula minuta strain RY4 was found to produce 2% PHA in biomass over a growth period of 96 h in a medium containing glucose, oleic acid, and Tween 60. The PHAs produced by Rhodotorula minuta strain RY4 were characterized using infrared spectroscopy and nuclear magnetic resonance (1 H and 13 C NMR) spectroscopy, revealing the presence of poly(3-hydroxybutyrate-co-3-hydroxyvalerate) (PHBV). This study demonstrated the ability of wild-type yeasts to produce PHAs, which is significant given the limited understanding of PHA biosynthesis in these organisms. Additionally, Thu et al.^5^ isolated a halophilic yeast strain, Pichia kudriavzevii TSLS24, from sediment samples in Vietnam. This strain was found to produce poly(3-hydroxybutyrate) (PHB) with a content of 43.4% and a concentration of 1.8 g/L after 7 days of cultivation in a medium containing glucose and yeast extract. The PHB produced by this strain demonstrated excellent biodegradability, with a degradation rate of 28% after 28 days of incubation in seawater.

Beyond wild-type strains, the synthesis of PHAs in genetically modified yeasts has garnered significant interest due to the potential for sustainable and high-yield production of biodegradable polymers. Saccharomyces cerevisiae has been a primary focus of research in this area due to its well-characterized genetics and established fermentation processes. Engineering efforts have included the introduction of bacterial PHA biosynthesis genes, leading to successful production of polyhydroxybutyrate (PHB)^7^. Further innovations targeted the peroxisome for PHA synthesis by utilizing intermediates of fatty acid β-oxidation, which enhanced production efficiency^8^. Recent advancements have enabled the production of D-lactic acid-containing PHA polymers in S. cerevisiae, illustrating the strain’s versatility^9^. A comparative study by Abd-El-Haleem et al.^10^ demonstrated PHA biosynthesis in both S. cerevisiae and Kloeckera spp. equipped with the phaABC operon from Ralstonia eutropha. This operon includes genes encoding β-ketothiolase, NADPH-linked acetoacetyl-CoA reductase, and PHA synthase. The study revealed that the non-conventional transgenic strain KY1/PHA accumulated up to 7.06% of the copolymer poly-(3-hydroxybutyrate-co-poly-3-hydroxyvalerate) (PHV), highlighting the capability of genetically modified yeasts to produce PHAs with tailored properties^10^.

In addition to S. cerevisiae, other yeast strains such as Yarrowia lipolytica have shown promise in PHA production due to their natural lipid accumulation capabilities. Gao et al.^11^ successfully engineered Y. lipolytica to produce medium-chain-length PHAs by introducing specific PHA synthase genes, broadening the range of potential applications. Modifications to the β-oxidation multifunctional protein in Y. lipolytica significantly increased PHA content and altered monomer composition, highlighting the importance of metabolic engineering^12^. Moreover, the utilization of industrial by-products like vinasse for PHA production in yeasts such as Haloferax mediterranei demonstrates a sustainable approach to biopolymer synthesis^13^.

Despite the strides made in bacterial PHA production, the exploration of yeast in this realm remains relatively limited. Our investigation began with a comprehensive screening of PHA-producing wild-type yeasts. Employing a diverse set of analytical techniques—including Nile red staining for visual confirmation, transmission electron microscopy (TEM) for high-resolution imaging, Fourier-transform infrared (FTIR) spectroscopy for chemical identification, and thorough molecular genetic analyses—we successfully identified PHA accumulation within the *Hanseniaspora valbyensis* DMG-2 yeast strain. With this significant discovery, our attention turned to unraveling the genetic mechanisms governing PHA biosynthesis in this specific yeast species. Recognizing the importance of this endeavor, we embarked on a targeted exploration to characterize the PHA biosynthesis genes within the genome of *Hanseniaspora valbyensis* DMG-2. To achieve this, we utilized molecular genetic tools and methodologies. Central to our approach was the construction and subsequent screening of a genomic library tailored to the *Hanseniaspora valbyensis* DMG-2 yeast strain. This meticulous process allowed us to sift through the yeast’s genetic makeup, isolating and identifying putative genes associated with PHA biosynthesis. Notably, this aspect of our study represents a pioneering effort, as previous literature had lacked investigations into the genetic machinery responsible for polyhydroxyalkanoates synthesis in this yeast species.

## Results

### Screening and Phylogenetic Analysis of PHA-Accumulating Yeast Isolates

**Figure 1:**
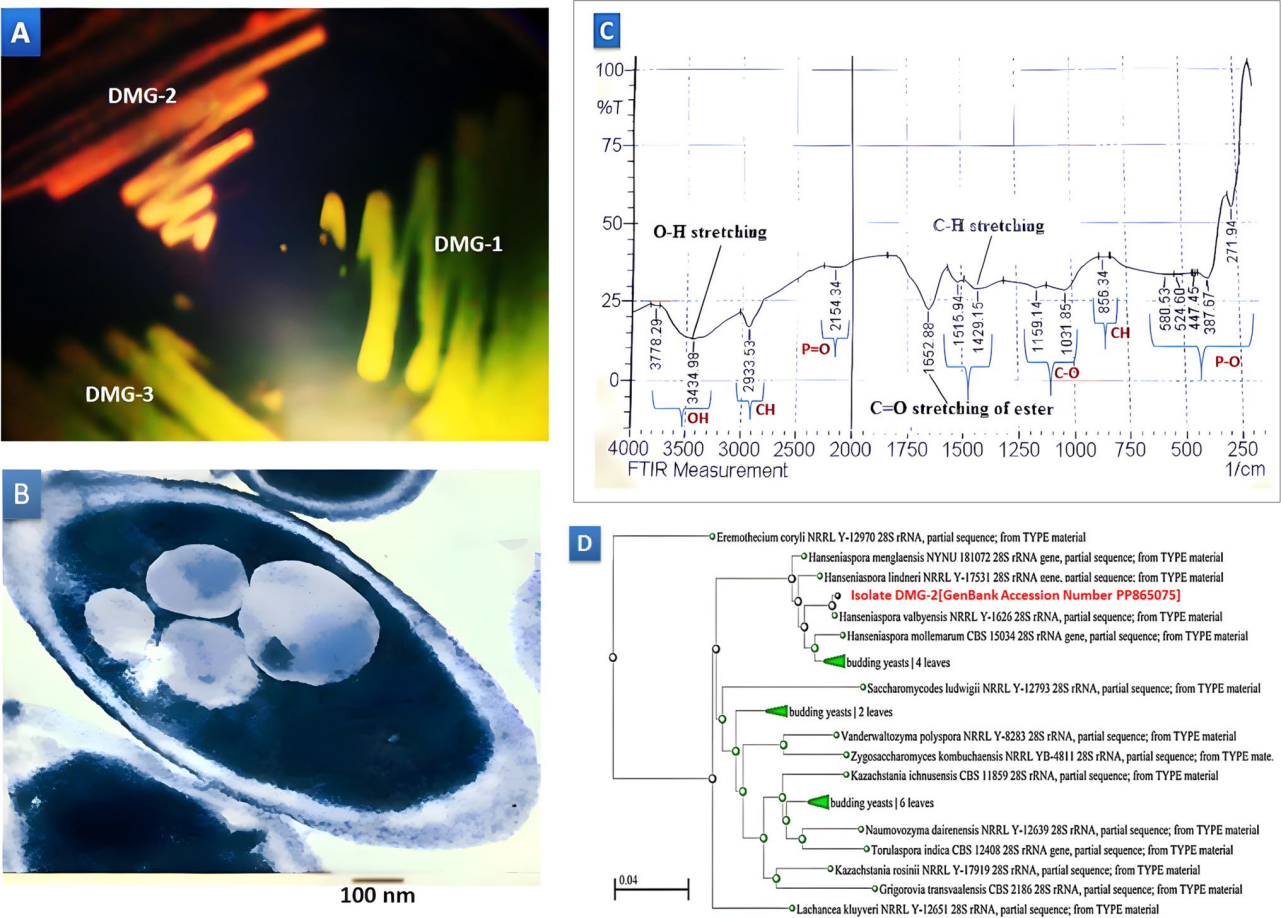
Presents key findings supporting the polyhydroxyalkanoate (PHA) accumulation capabilities of isolate DMG-2. **(A)** The Nile Red staining assay under UV light highlights DMG-2’s significant red fluorescence, indicative of substantial PHA accumulation compared to other isolates like DMG-1 and DMG-3, which show no red fluorescence. **(B)** Electron micrograph images of DMG-2 cells reveal numerous electron-dense PHA granules within the cytoplasm, confirming intracellular storage of these biopolymers. The scale bar denotes a size reference of 100 nm, emphasizing the distribution and abundance of the granules. **(C)** The FTIR spectrum of DMG-2 inclusion bodies displays absorption peaks, suggesting a complex composition potentially comprising PHAs and polyphosphates (PP). **(D)** The phylogenetic tree constructed using the 28 S ribosomal DNA (rDNA) sequence of DMG-2 depicts its evolutionary relationship within the fungal kingdom. DMG-2 clusters within a distinct clade closely related to Hanseniaspora species, underscoring its unique taxonomic position and evolutionary divergence from other yeast species.

The study commenced with a thorough screening process to identify yeast isolates capable of accumulating polyhydroxyalkanoates (PHAs). To detect PHAs and various lipid compounds, the Nile red staining assay was employed due to its versatility. Among the numerous wild-type yeast strains examined under UV transillumination, the isolate DMG-2 was notable for its distinct red fluorescence, indicating PHA accumulation (Fig. 1A). Transmission electron microscopy (TEM) analysis of the DMG-2 isolate, validated to accumulate PHAs via the Nile red assay, revealed detailed cell morphology. The cells display an elongated, oval shape typical of yeast. Visible within the cells are electron-dense granules, likely representing storage materials such as PHA granules or polyphosphate intracellular inclusions. The cytoplasm appears uniformly electron-lucent. The scale bar indicates 100 nm (Fig. 1B).

The FTIR spectrum of the polymer extracted from isolate DMG-2, identified as PHA co-polyphosphate (Fig. 1C), reveals characteristic peaks at 3778 cm^−1^ and 3434 cm^−1^, denoting hydrogen-bonded O-H stretching vibrations. These peaks potentially originate from hydroxyl groups present in both PHA and polyphosphate. The peak at 2933 cm^−1^ signifies C-H stretching vibrations, likely emanating from alkyl groups within the PHA structure. The presence of polyphosphate is discerned by the peak at 2154 cm^−1^, representing stretching vibrations of P = O bonds. Peaks at 1625 cm^−1^, 1515 cm^−1^, and 1429 cm^−1^ suggest C = O stretching vibrations, possibly from carbonyl groups in both PHA and polyphosphate. Additionally, peaks at 1159 cm^−1^ and 1031 cm^−1^ indicate C-O stretching vibrations, potentially from ether or ester groups in PHA and polyphosphate, respectively. The peak at 856 cm-1 suggests C-H bending vibrations, likely from alkyl groups in PHA. The P-O stretching is indicated by peaks at 580.53 cm^−1^, 477.45 cm^−1^, 387.57 cm^−1^, and 271.94 cm^−1^. These features confirm the polymer’s composition as a polyhydroxyalkanoate co-polyphosphate.

The BLASTN search against the 28 S ribosomal RNA sequences from fungal type and reference material identified multiple significant alignments for the DMG-2 sequence, which were 221 base pairs in length. The top matches included *Hanseniaspora valbyensis* NRRL Y-1626 with a maximum score of 403 and 99.55% identity, *Hanseniaspora menglaensis* NYNU 181,072 with a score of 370 and 96.85% identity, and *Hanseniaspora mollemarum* CBS 15,034 with a score of 368 and 96.83% identity. Other notable alignments included *Hanseniaspora lindneri*, *Hanseniaspora jakobsenii*, *Hanseniaspora lachancei*, *Hanseniaspora uvarum*, *Hanseniaspora nectarophila*, *Hanseniaspora clermontiae*, and *Hanseniaspora opuntiae*, all of which showed significant scores and high identity percentages ranging from 91.40 to 95.93%. Phylogenetic analysis of the DMG-2 isolate (Fig. 1D), based on the 28 S rDNA sequence, positioned it within the fungal lineage. Using NCBI’s distance tree tools and the neighbor-joining method, the tree revealed that DMG-2 clusters with *Hanseniaspora valbyensis* NRRL Y-1626, albeit with some evolutionary divergence. This analysis also highlighted DMG-2’s distinct evolutionary distance from other budding yeast species, confirming its unique taxonomic placement. Consequently, DMG-2 is identified as *Hanseniaspora valbyensis* strain DMG-2.

### Cloning of Putative PHA Biosynthesis Genes from DMG-2 Yeast

**Figure 2:**
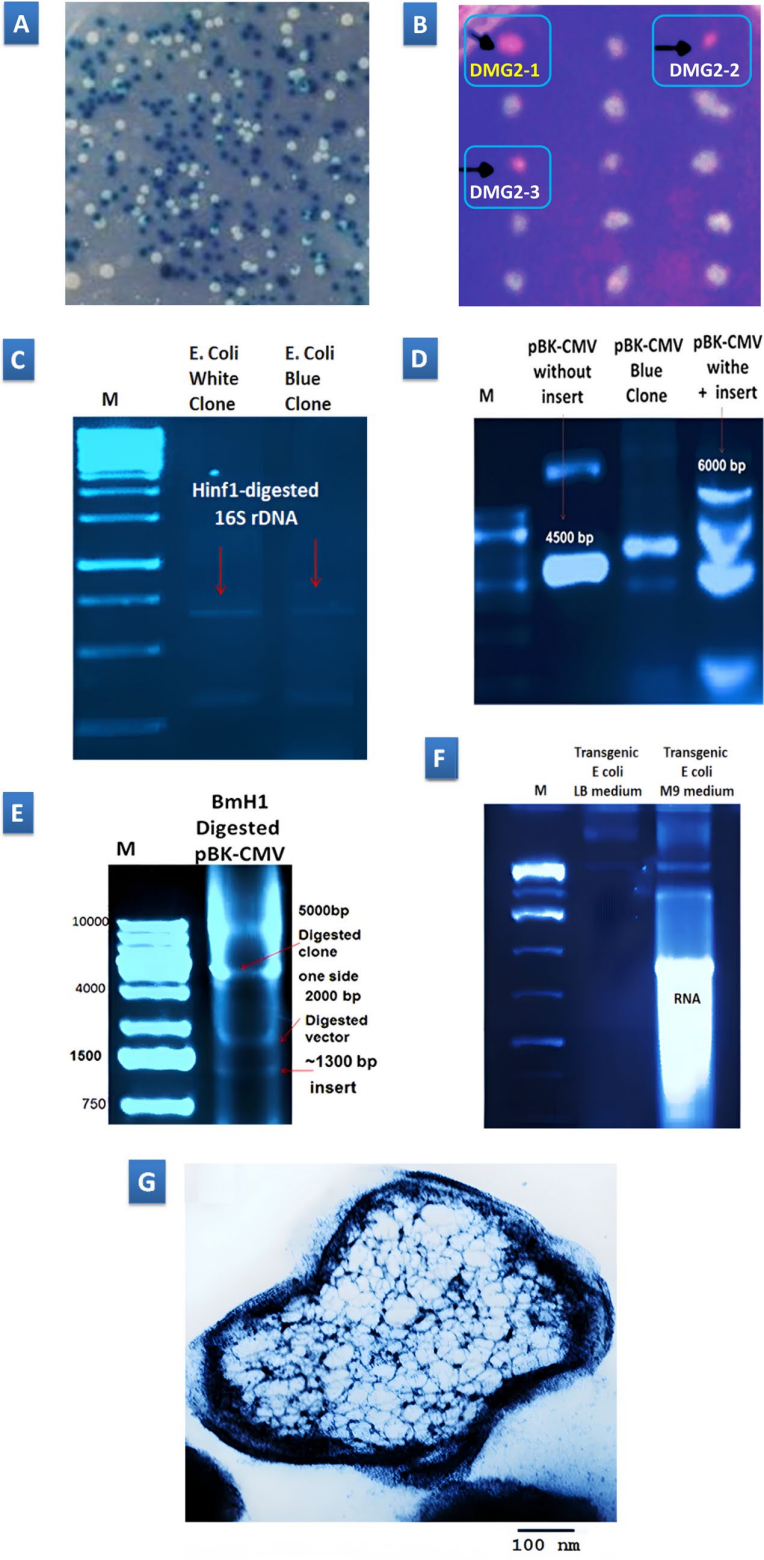
Illustrates the process of identifying genes for polyhydroxyalkanoate (PHA) production in yeast strain DMG-2. **(A)** The white/blue colony color assay is employed to identify clones containing DMG-2 DNA inserts. **(B)** Confirmation of PHA production is achieved through the Nile red fluorescence assay, with positive clones exhibiting red fluorescence, including DMG2-1 (selected for further analysis), DMG2-2, and DMG2-3. **(C)** Restriction fragment length polymorphism (RFLP) analysis of the 16 S ribosomal RNA gene distinguishes between *E. coli* and yeast cells, ensuring no contamination. **(D)** Gel electrophoresis verifies the presence of DNA fragments from wild-type strain DMG-2 inserted into plasmids via cutting with a *Bam*H1 restriction enzyme. **(E)** Gel electrophoresis confirms the presence of the inserted fragment within the vector backbone of transgenic *E. coli* DMG2-1. **(F)** Gel electrophoresis assesses promoter activity by comparing plasmid DNA profiles of transgenic *E. coli* strain DMG2-1 grown in different media (M9 and LB). **(G)** Transmission electron microscopy visualizes PHA granules within transformed *E. coli* DMG2-1 cells, confirming successful gene transfer and expression.

The research endeavors focused on the isolation and in-depth characterization of putative polyhydroxyalkanoate (PHA) biosynthesis genes derived from wild-type DMG-2 yeast, findings intricately intertwined with the comprehensive data delineated in Fig. 2A-G. Commencing with the construction of a genomic library through partial *Bam*H1 digestion and subsequent ligation into the *Bam*H1-linearized pBK-CMV vector, the study meticulously identified colonies potentially housing PHA genes, employing rigorous screening assays including blue/white selection and Nile Red staining (Fig. 2A and B). Further validation via restriction mapping and 16 S rDNA RFLP affirmed the purity of selected colonies (Fig. 2C-D), laying the groundwork for subsequent molecular dissections. Gel electrophoresis emerged as a pivotal tool in delineating clones carrying inserts, unveiling a ~ 1.3 kb *Bam*H1 fragment speculated to encompass the PHA genes (Fig. 2E), subsequently subcloned into the pUC18 vector for downstream molecular analyses. The discernible amplification of RNA yields within the pBK-CMV clone when cultured in production media (M9) as opposed to LB media underscored the functional prowess of the expression vector’s promoter, indicative of heightened protein expression potential (Fig. 2F).

The combined findings suggest a promising outlook for increased protein expression and enhanced accumulation of polyhydroxyalkanoates (PHA) within the clone. This is underscored by the vivid transmission electron microscopy (TEM) depiction of PHA inclusions following a 72-hour culture on M9 media (Fig. 2G). The TEM image portrays a thin section of a genetically modified *E. coli* cell containing PHA genes sourced from strain DMG-2. The cell displays typical features of *E. coli*, including a well-defined, electron-dense outer membrane and a discernible cell wall. Within the cytoplasm, numerous electron-dense granules are visible, likely representing PHA inclusions. These granules manifest as dark, irregularly shaped structures dispersed throughout the cytoplasmic matrix. The distribution and abundance of these PHA granules indicate successful expression and accumulation of polyhydroxyalkanoates within the transgenic *E. coli* cells. The cytoplasm itself appears electron-lucent, serving as a contrasting background that accentuates the dense PHA granules. This visualization unequivocally validates the efficient integration and functionality of the PHA biosynthetic pathway genes from strain DMG-2 in the *E. coli* host, culminating in the formation of intracellular PHA granules. The scale bar in the image denotes a magnification level conducive to detailed examination of these intracellular structures, confirming their size and morphology consistent with established PHA granules.

### Decoding of Open Reading Frame 1 (ORF1): Predicted Protein sequence

**Figure 3:**
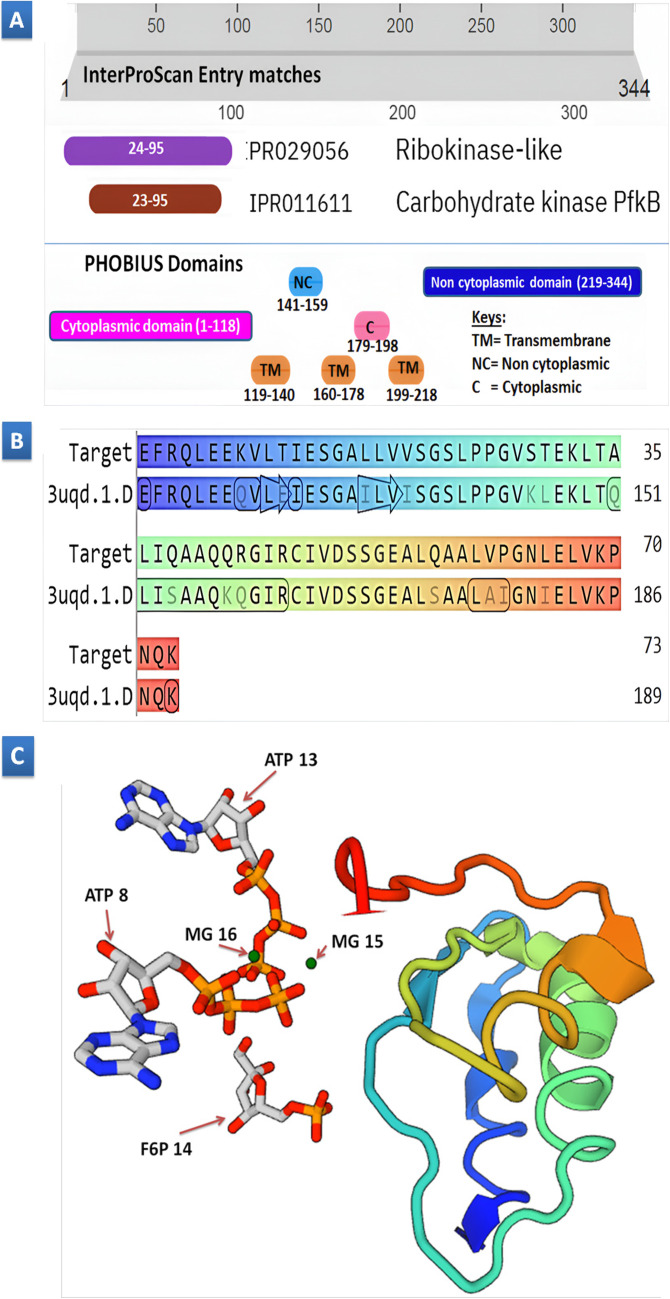
Provides structural insights into the cytoplasmic domain of the multifunctional ORF1 protein. **(A)** InterProScan and Phobius domain annotations depict the entire ORF1 protein sequence (344 residues). **(B)** Comparative sequence alignment of protein structures demonstrates the alignment between the predicted model from Swiss-modeling (based on template 3uqd) and the cytoplasmic domain predicted within ORF1 (amino acids 23–95). **(C)** The 3D structure of the cytoplasmic domain within ORF1, as modeled by Swiss-modeling, utilizes the phosphofructokinase-2 enzyme (PDB ID: 3uqd) as a template. The presence of ligands ATP, F6P, and MG is also indicated in the modeled structure.

Upon M13-PCR primer sequence alignment and elimination of the 1379-bp Victor contamination, the NCBI ORF finder tool revealed a single open reading frame (ORF1) within the DNA sequence of DMG-2 insert. ORF1 spans 1035 nucleotides, encoding a 344-amino acid protein. Translation used the standard genetic code with ATG as the sole start codon, excluding nested ORFs during analysis. InterPro suggests ORF1’s potential involvement in sugar metabolism due to homology with the carbohydrate kinase PfkB family (residues 23–95) and the ribokinase-like superfamily (residues 7-105), known for phosphorylating sugar molecules. PHOBIUS analysis of transmembrane regions suggests ORF1 likely resides within the cell membrane, with alternating transmembrane helices (residues 119–140, 160–178, 199–218) separating cytoplasmic domains (residues 1-118, 179–198) from non-cytoplasmic domains (residues 141–159, 219–344), facing the extracellular space. This structural arrangement implies potential membrane tethering, with functional domains poised for interaction with molecules on both sides, supporting its sugar metabolism role as suggested by homologies to PfkB and ribokinase domains (Fig. 3A).

BLASTAlignPeptide and PHOBIUS analyses identified a multifunctional protein with distinct domains (Table 1). The transmembrane domain (residues 119–140) showed high similarity to a phosphohydrolase from Sporosarcina sp. P19, suggesting a role in phosphate hydrolysis. The extracellular domain (residues 141–159) lacked significant hits, but other domains displayed similarities: glycosyltransferase protein from Hirsutella rhossiliensis (residues 160–178) in the transmembrane region, diverse organisms in the cytoplasmic domain (residues 179–198) with roles in meiosis and phosphorylation, and a putative salt-induced outer membrane protein from Enterobacter sp. BIGb0383 in the outer membrane domain (residues 199–218). The non-cytoplasmic domain (residues 219–344) showed moderate similarity to ATP synthase gamma chain and geranylgeranyl diphosphate synthase, suggesting potential roles in ATP synthesis or geranylgeranyl diphosphate synthesis.

**Table 1:** Domain annotations and functional predictions of the multifunctional protein identified by BLASTAlignPeptide and PHOBIUS analyses.

Residue Range	Domain Type	Function	Homology/Similarity
1-118	N-terminal Cytoplasmic	Involved in sugar metabolism	Carbohydrate kinase PfkB family (residues 23–95)
119–140	First Transmembrane Helix	Possible membrane transport or signaling	Phosphohydrolase from Sporosarcina sp. P19 (77% identity, 92% similarity)
141–159	First Non-cytoplasmic	Likely interacts with extracellular molecules	No significant hits identified
160–178	Second Transmembrane Helix	Likely membrane transport or signaling	Glycosyltransferase protein from Hirsutella rhossiliensis (76% identity, 76% similarity)
179–198	Second Cytoplasmic	Potential role in meiosis and phosphorylation	Similarity to proteins involved in meiosis and phosphorylation
199–218	Third Transmembrane Helix	Likely membrane transport or signaling	Putative salt-induced outer membrane protein from Enterobacter sp. BIGb0383 (84% identity, 89% similarity)
219–344	Third Non-cytoplasmic	Potential role in ATP synthesis or geranylgeranyl diphosphate synthesis	ATP synthase gamma chain (38.1 bits, 40% identity, 51% positives); Geranylgeranyl diphosphate synthase (35.8 bits, 31% identity, 49% positives)

The ORF1 protein sequence indicates the presence of a fragment of an ATP synthase protein. Specifically, it contains a motif corresponding to the Walker B motif, which spans residues 250 to 256 and is represented by the sequence “IVLDWTR”. This motif plays a crucial role in coordinating magnesium ions, essential for ATP hydrolysis and subsequent conversion to ADP and inorganic phosphate. Additionally, the sequence extends beyond residue 344, suggesting the involvement of further residues in contributing to the catalytic and structural functions of ATP synthase.

PROSITE analysis revealed a multitude of functional motifs within the ORF1 protein sequence, hinting at potential post-translational modifications that could influence its stability, localization, and interactions. Notably, an N-glycosylation site was identified at position 219 with the pattern N[P][ST], presenting the sequence NATA. Additionally, a cAMP- and cGMP-dependent protein kinase phosphorylation site was found at position 179, following the pattern [RK]{2}.[ST], with the sequence KKDS. Moreover, several protein kinase C phosphorylation sites were detected: at positions 52 and 150 with the pattern [ST].[RK], presenting the sequences TEK and TKR, respectively. Furthermore, casein kinase II phosphorylation sites were identified at multiple positions: 20, 73, 221, 228, 268, 298, and 305, with the pattern [ST].{2}[DE], presenting the sequences TDDE, SSGE, TADD, TVMD, TPDD, SGDD, and SIVD, respectively. Additionally, N-myristoylation sites were predicted at positions 12, 183, 212, and 261 with the pattern G[EDRKHPFYW].{2}[STAGCN][P], presenting the sequences GLSSGA, GTVWSL, GGVIAA, and GLVDGS, respectively. An amidation site was also identified at position 236 with the pattern .G[RK][RK], presenting the sequence GGRR. These motifs communally suggest that the ORF1 protein may undergo various post-translational modifications, such as glycosylation and myristoylation, which could significantly impact its stability, localization, and interactions.

The PROFsec analysis predicts that the ORF1 protein has a diverse secondary structure, with approximately 40.7% alpha helices and 18.02% beta sheets, indicating a complex folding pattern. Furthermore, solvent accessibility analysis suggests a balanced distribution of exposed (55.81%) and buried residues (44.19%), reflecting both defined structural elements and flexible regions for potential interaction with other molecules. The PSI-BLAST alignment reveals significant homology with several proteins: pdb1DGM_A (Adenosine Kinase from Toxoplasma gondii) with 21% sequence identity and 35% similarity over 98 aligned residues, pdb1LHR_A (Pyridoxal Kinase Complexed with ATP) with 15% identity and 36% similarity over 103 aligned residues, pdb1RFU_A (Pyridoxal Kinase Complexed with ADP and PLP) with identical identity, similarity, alignment length, and BLAST score to pdb1LHR_A, pdb1RK2_B, pdb1RKA_A, and pdb1RKD_A (*E. coli* Ribokinase variants) with 21% identity and 44% similarity over 106 aligned residues, and pdb1TYY_B (Aminoimidazole Riboside Kinase from Salmonella enterica) with 17% identity and 35% similarity over 116 aligned residues. These findings suggest structural and functional similarities between the ORF1 protein and these homologous enzymes, particularly in domains relevant to ATP binding and metabolism.

In the context of a larger protein translated from ORF1 (344 residues), the identified template (3uqd.1.D) for the segment corresponding to amino acids 23–95 provides valuable structural insights using Swiss-modeling (Fig. 3B and C). This template, originating from Escherichia coli phosphofructokinase-2, exhibits high structural similarity (0.85 identity, 80.82% coverage). The homo-tetrameric structure determined by X-ray crystallography suggests a potential functional role for protein oligomerization within this domain of the larger protein. Significantly, the template reveals bound ATP (likely at the nucleotide binding site) and F6P (Fructose-6-phosphate), a substrate for phosphofructokinase-2. While the specific interactions are not available here, this suggests a potential substrate-bound conformation for this segment. The presence of magnesium (MG) ions further strengthens the model as these often play a crucial role in protein stability and enzyme function. No hits were predicted using the Swiss-Modeling tools for the other transmembrane, cytoplasmic, and non-cytoplasmic segments identified by PHOBIUS.

### Decoding of ORF1: DNA sequence

**Figure 4:**
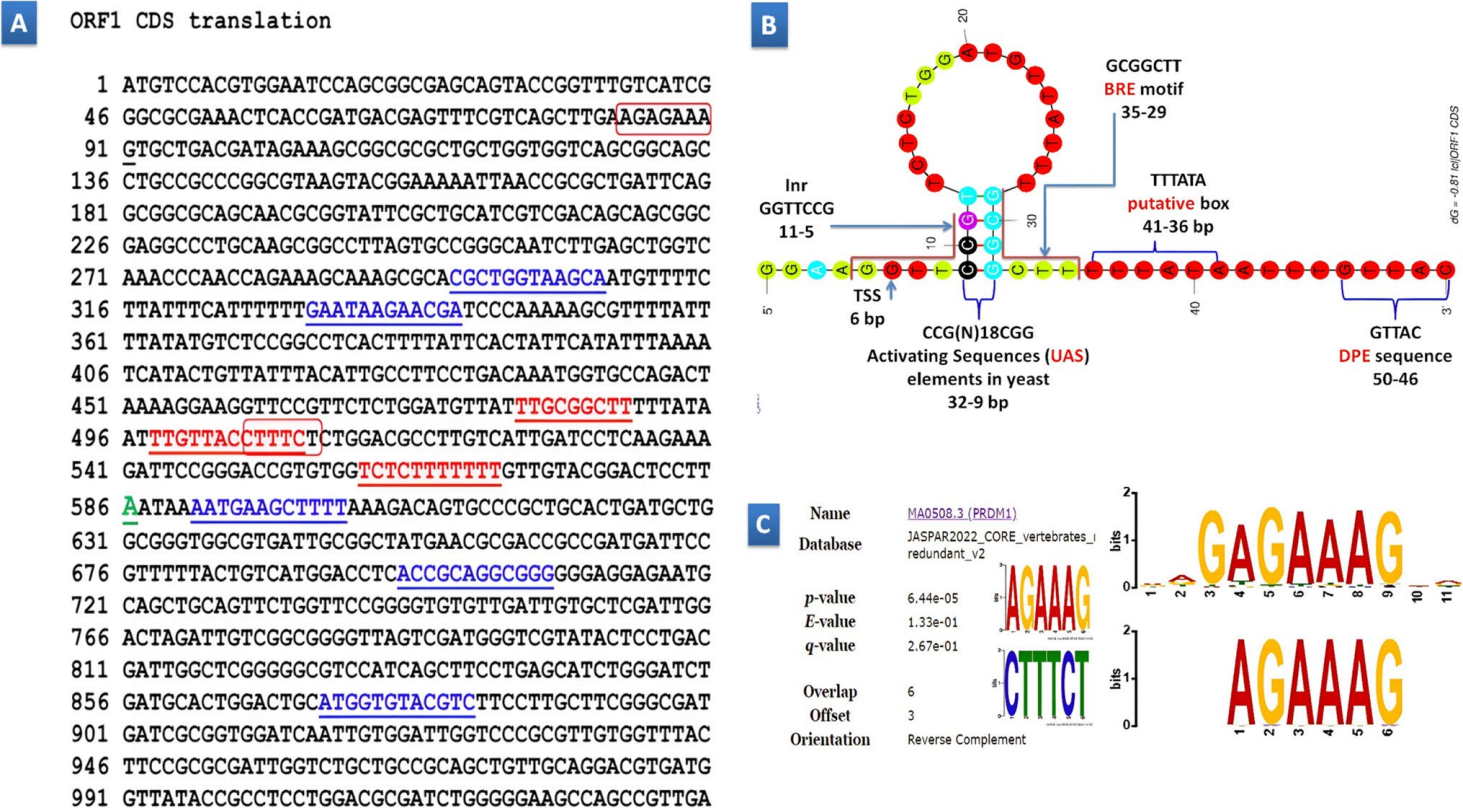
Illustrates the genomic landscape of ORF1 (1035 bp, GenBank AC PP869688), highlighting key regulatory elements and functional motifs. **(A)** The depiction includes a potential polyadenylation site at nucleotide 586 highlighted in green, along with donor and acceptor splice sites crucial for mRNA processing marked with blue and underlines, respectively. Five TFBS motifs identified within the sequence are marked with red and underlines, with the conserved motif “AGAAAG/CTTTCT” enclosed within red boxes as identified by MEME analysis. **(B)** The predicted structural model of the ORF1 promoter region reveals a complex architecture featuring a 26-base external loop, a single closing helix, and stabilizing elements. Functional motifs such as TSS, Inr, BRE, TATA box, and DPE are strategically positioned, suggesting roles in transcription initiation and complex assembly. UAS elements identified in yeast underscore the regulatory landscape. **(C)** The MEME positive and complement discovered motif AGAAAG/CTTTCT is aligned with the top hit motif profile MA0508.3 representing the PRDM1 transcription factor. A significant match (p-value: 6.44002e-05) with an optimal offset of 3 nucleotides offers insights into regulatory associations and genetic mechanisms.

As shown in Fig. 4A, the POLYAH tool has pinpointed a potential polyadenylation site within the 1035-base-pair sequence provided, precisely at position 586. This site carries a polyadenylation likelihood score of 2.23, suggesting its candidacy for polyadenylate tail cleavage and addition to mRNA transcripts in the vicinity of position 586. Meanwhile, the FSPLICE 1.0 analysis scrutinized genomic DNA sequences from two organisms, Schizosaccharomyces pombe (S. pombe) and Saccharomyces cerevisiae (S. cerevisiae), unveiling crucial splice sites vital for mRNA processing. In the S. pombe sequence, four potential donor (GT) sites and one acceptor (AG) site were discerned. The highest scoring donor site, tallying a score of 10.30, resided at position 301 (“cgctgGTaagca”), while the acceptor site, scoring 6.92, manifested at position 597 (“aatgaAGctttt”). Conversely, in the S. cerevisiae sequence, three donor sites and two acceptor sites were predicted. The most significant donor site, scoring 9.46, appeared at position 876 (“atggtGTacgtc”), with acceptor sites scoring 5.47 and 7.25 found at positions 336 (“gaataAGaacga”) and 703 (“accgcAGgcggg”), respectively. These revelations offer valuable insights into potential splice junctions pivotal for mRNA maturation in these organisms, laying the groundwork for further explorations into gene expression regulation and plausible connections to metabolic pathways such as polyhydroxyalkanoates synthesis or other cellular processes.

The Nsite Version 6.2014 analysis delved into a 1035-base-pair test sequence, uncovering five distinct TFBS motifs. These shed light on potential transcriptional regulatory elements influencing metabolic pathways, including polyhydroxyalkanoates (PHA) synthesis. Among these motifs, the maize gene cyPPDK1, encoding a cytosolic pyruvate orthophosphate dikinase, showcased a box e TFBS motif “AAAAAAGAGA” at position 568 on the negative (-) strand (TCTCTTTTTT), indirectly affecting carbon metabolism pertinent to PHA precursor synthesis. Similarly, Arabidopsis’ PCbox1 motif “GAAaGGTAACAA/ TTGTTACCtTTC " at position 509, with one mismatch, may modulate genes intersecting with PHA precursor pathways. Pinus sylvestris motifs corresponding to TFBSs AT-1 and AT-2a on the positive (+) strand at positions 403 and 490, respectively, hint at regulatory roles in carbohydrate metabolism, potentially influencing PHA precursor availability. Furthermore, the rice gene Amy3E’s motif “TTGCGGCTT” at position 481 may influence starch metabolism, indirectly affecting carbon fluxes pertinent to PHA precursor biosynthesis. Although these TFBS motifs do not directly correlate with PHA synthesis, they suggest regulatory networks intersecting with carbon metabolism, potentially influencing precursor availability or pathway regulation for PHA biosynthesis.

The CpGFinder analysis identified two CpG islands within the provided DNA sequence: the first spanning from position 8 to 252 on the positive strand, comprising 32 CpG dinucleotides with a GC content of 61.6%, and the second ranging from position 632 to 1012, also on the positive strand, containing 34 CpG dinucleotides with a GC content of 58.0%. Subsequent protein analysis unraveled that ORF1, located within this sequence, encodes a 344-amino acid protein likely residing within the cell membrane. This protein exhibits homology to carbohydrate kinase and ribokinase-like domains, suggesting its involvement in sugar metabolism. Additionally, distinct functional domains were identified—transmembrane, cytoplasmic, and outer membrane domains. Structural modeling further unveiled similarity to phosphofructokinase-2, indicating a potential role in substrate binding and oligomerization. These findings cooperatively underscore the regulatory significance of CpG islands and provide detailed insights into the multifunctional nature of the encoded protein, particularly in sugar metabolism and membrane-associated processes.

In silico analysis utilizing the Neural Network Promoter Prediction (NNPP) tool unearthed a putative promoter region (′3- GGAAGGTTCCGTTCTCTGGATGTTATTTGCGGCTTTTTATAATTTGTTAC-′5) on the reverse strand (positions 504 − 454) of the ORF1 DNA sequence with a high score (0.97), indicative of a robust candidate for a eukaryotic promoter. Key elements characteristic of eukaryotic promoters were observed: a putative TATAAA/ TTTATA box likely resides upstream (positions 42 − 36), a potential BRE motif (AAGCCGC/GCGGCTT, positions 11 − 5) flanks the TATAAA/ TTTATA box, the core promoter element Inr (CGGAACC/GGTTCCG, positions 35 − 29) overlaps the proposed transcription start site (TSS) at position 41 with the characteristic initiator “G”, and a putative DPE sequence (GTAAC/GTTAC, positions 50 − 46 is found downstream. Interestingly, the motif CGG(N)18CCG/CCG(N)18CGG at the positions 32 − 9 within the promoter bears significant resemblance to Upstream Activating Sequences (UAS) elements in yeast, particularly the Overlapping Replication Element (Kagawa 2010). The S. cerevisiae ORE consensus sequence features inverted CGG triplets separated by 15–18 nucleotides (CGG(N)15-18CCG), a recognition motif for C6 zinc cluster fungal transcriptional regulators like Gal4p^14^.

The predicted promoter region of ORF1, encompassing 50 nucleotides (Fig. 4B), underwent thorough analysis using the UNAFold Web Server employing the mfold algorithm at physiologically relevant conditions of 37 °C with 1.0 M Na + and 0.0 mM Mg++. The resultant most stable structural model, exhibiting a free energy of -0.81 kcal/mol, revealed a complex architecture characterized by an external loop comprising 26 single-stranded bases accompanied by a single closing helix. Notably, the stability of this structure was significantly bolstered by three stacks (C9-G32, C10-G31, G11-C30) with respective free energies of -1.84, -2.17, and − 0.59 kcal/mol, alongside a hairpin loop stabilized by the T12-G29 base pair, contributing 4.60 kcal/mol. Furthermore, the presence of a four-base-pair helix within the structure further reinforced its stability, with an associated free energy of -4.60 kcal/mol. The strategic positioning of sequence elements within the fold offers invaluable insights into their functional roles. Specifically, the transcription start site (TSS) and the initiator (Inr) element, located within the external loop, facilitate accessibility for transcription initiation factors. Adjacent to the structured helix, the TFIIB recognition element (BRE) motif potentially aids in stabilizing transcription factor binding. Moreover, the putative TATA box, positioned near the closing helix, suggests involvement in the assembly of the transcription complex. Conversely, the downstream promoter element (DPE) sequence, situated in a flexible, single-stranded region, likely plays a pivotal role in transcription factor recruitment. Additionally, the identification of Activating Sequences (UAS) elements in yeast, spanning positions 32 − 9, underscores the multifaceted regulatory landscape governing promoter dynamics and gene expression modulation.

The MEME analysis prominently identified the motif “AGAAAG”, characterized by a width of 6 nucleotides and found in five sequences with a compelling log-likelihood ratio of 42 and an E-value of 1.9e + 004. This motif, represented by the consensus sequence AGAAAG, suggests a conserved nucleotide pattern crucial for regulatory functions. In the Tomtom version 5.5.5 motif comparison analysis, the query motif “AGAAAG” was scrutinized against various motif databases, uncovering significant matches, notably the top hit with matrix profile MA0508.3 representing the PRDM1 transcription factor. MA0508.3, part of the C2H2 zinc finger factors class within JASPAR’s CORE collection, derived from ChIP-seq data for Homo sapiens, displayed a distinct nucleotide preference pattern (Fig. 4C). The alignment with the query motif revealed a statistically significant match (p-value: 6.44002e-05) with an optimal offset of 3 nucleotides, indicating a specific positional relationship. This detailed analysis offers crucial insights into potential regulatory associations and genetic mechanisms, contributing significantly to our understanding of biological processes.

### Spectroscopic analysis of the biopolymer extracted from Transgenic E coli DGM2-1

**Figure 5:**
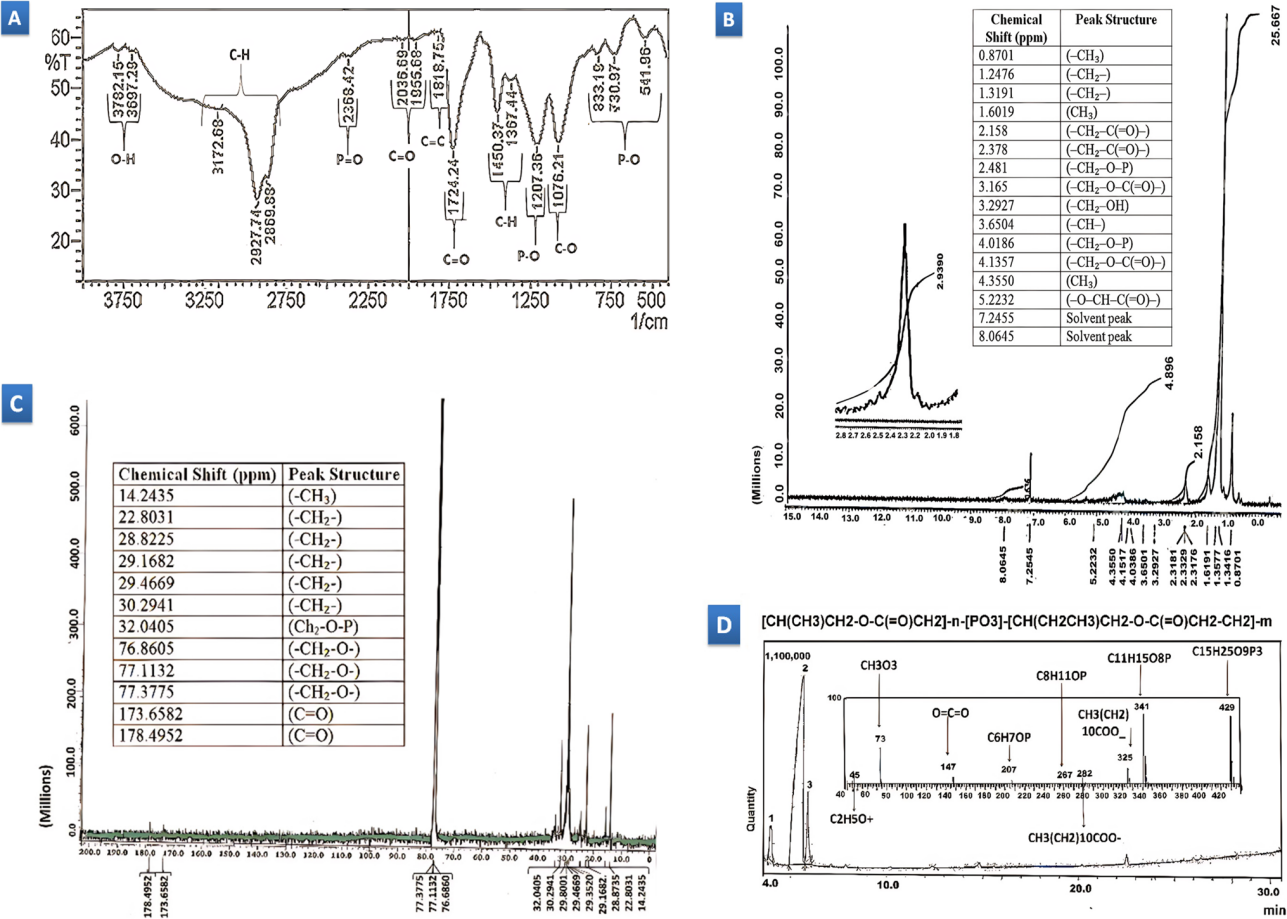
Provides a comprehensive analysis of the biopolymer’s structure extracted from the transgenic *E. coli* clone DMG2- using a variety of spectroscopic techniques. **(A)** The FTIR spectrum displays characteristic peaks representing functional groups such as hydroxyl, aliphatic hydrocarbons, phosphates, and esters, indicating the presence of polyhydroxyalkanoates (PHAs) and polyphosphates. **(B)** Detailed information on hydrogen environments within the polymer is revealed by the ^1^H NMR spectrum, confirming the presence of terminal methyl groups, backbone methylene groups, and those adjacent to specific linkages, supporting the existence of both PHB and PHV units. **(C)** The ^13^C NMR spectrum further clarifies the carbon environment, assigning peaks to specific carbon types (methyl, methylene and carbonyl) and their positions within the structure, highlighting the complex connectivity of carbon atoms. **(D)** The GC-MS chromatogram exhibits three distinct peaks, and the proposed structure illustrates a copolymer comprising PHB and PHV units linked by ester bonds. Fragmentation analysis identifies the location of phosphate groups, potentially attached to the biopolymer fragment in the chromatogram peak 3.

A thorough examination utilizing Fourier-Transform Infrared (FTIR), Proton Nuclear Magnetic Resonance (1 H NMR), Carbon Nuclear Magnetic Resonance (13 C NMR), and Gas Chromatography-Mass Spectrometry (GC-MS) elucidates the intricate structure of a biopolymer, likely a copolymer comprised of polyhydroxyalkanoates (PHAs) and polyphosphates. FTIR spectroscopy **(**Fig. 5A**)** unveils broad peaks at 3782 cm-1 and 3697 cm-1, indicating hydrogen-bonded O-H stretching vibrations from hydroxyl groups present in both PHA and polyphosphate. Peaks at 3172.68 cm-1, 2927 cm-1, and 2869 cm-1 signify C-H stretching vibrations from alkyl groups in the PHA backbone. Peaks at 2368 cm-1, 1207 cm-1, and 730 cm-1 suggest the presence of polyphosphate through P = O, P-O, and P-O-P stretching vibrations, respectively. C = O stretching vibrations from ester carbonyl groups in PHA are observed at 2036 cm-1, 1955 cm-1, and 1724 cm-1. Peaks at 1818 cm-1 and 1450 cm-1 indicate C = C and C-H bending vibrations, respectively. Additional peaks at 1367 cm-1 and 1076 cm-1 suggest C-H bending and C-O stretching vibrations from methyl and hydroxyl/ether groups in PHA and polyphosphate. The peak at 833 cm-1 signifies C-H bending vibrations from alkyl groups in the PHA structure, while the peak at 514 cm-1 corresponds to stretching vibrations of P-O bonds in polyphosphate.

1 H NMR spectroscopy confirms the presence of PHA and polyphosphate segments (Fig. 5B). Peaks at 0.8701 ppm, 1.2476 ppm, and 1.3191 ppm denote terminal methyl groups (-CH₃) and methylene groups (-CH₂–) in the polymer backbone of PHA. The peak at 1.6019 ppm suggests methylene groups adjacent to electronegative atoms or groups. Peaks at 2.158 ppm and 2.378 ppm indicate methylene protons adjacent to carbonyl groups (-CH₂–C(= O)–) in ester linkages of PHA. Methylene groups adjacent to oxygen atoms in ester linkages are observed at 3.165 ppm, while the peak at 3.2927 ppm is associated with methylene groups adjacent to hydroxyl groups (-CH₂–OH). The signal at 3.6504 ppm likely arises from methine protons (-CH–) adjacent to ester linkages. Peaks at 4.0186 ppm and 4.1357 ppm represent protons on carbons adjacent to phosphate groups (-CH₂–O–P) and methylene protons adjacent to ester and hydroxyl groups, respectively. The 4.3550 ppm peak suggests methylene groups adjacent to both ester and phosphate groups. The peak at 5.2232 ppm corresponds to protons on carbons adjacent to both oxygen and carbonyl groups (-O–CH–C(= O)–).

13 C NMR spectroscopy provides further details about PHA segments and confirms ester functionalities (Fig. 5C). The peak at 14.2435 ppm corresponds to terminal methyl carbons (-CH₃) in PHB and PHV segments, while the peak at 22.8031 ppm represents methylene carbons (-CH₂–) adjacent to these terminal methyl groups. Peaks between 28.8225 ppm and 30.2941 ppm denote repeating methylene units in the PHA backbone. A peak at 32.0405 ppm suggests methylene carbons adjacent to phosphate groups or distinct structural features within the PHB or PHV segments. Peaks at 76.86 ppm, 77.11 ppm, and 77.38 ppm denote methylene carbons adjacent to oxygen atoms (-CH₂-O-) in ester linkages, while peaks at 173.66 ppm and 178.50 ppm confirm ester functionalities by indicating carbonyl carbons (C = O) within the ester groups of the polymer chain.

A comprehensive GC-MS analysis reveals the biopolymer’s structure (Fig. 5D). Three distinct peaks emerge at retention times: 3.75–4.25 min (peak 1), 4.85–5.84 min (peak 2), and 5.79–6.31 min (peak 3). Peaks 1 and 2 potentially correspond to PHA monomers (PHB or PHV, or a combined PHBV peak) based on their retention times. Fragmentation patterns from these peaks are crucial for definitive identification. Peak 3, with a later retention time, is unlikely pure polyphosphate due to its fragmentation behavior. However, it could be a biopolymer fragment containing a phosphate group attached to the PHA segment. Analyzing fragmentation patterns, particularly for the pentamethylsilyl (PMSi) derivatized biopolymer, provides a window into its intricate structure. The fragmentation patterns observed across a range of m/z values offer valuable clues about the building blocks, potential linkages, and overall complexity of the polymer. The mass spectra of the pentamethylsilyl (PMSi) derivatized biopolymer exhibit characteristic fragmentation peaks at specific m/z values, including 45, 73, 147, 207, 267, 282, 325, 341, and 429.

The presence of a peak at m/z 45 signifies fragments like C2H5O+, indicative of simple hydroxyalkanoate units. This initial observation suggests the presence of basic building blocks associated with polyhydroxyalkanoates (PHAs) within the biopolymer. These PHAs likely constitute the fundamental structural framework of the polymer. The peak observed at m/z 73 likely corresponds to oxygen-containing fragments like CH3O3​ (trimethylsilyl). This finding suggests the presence of oxygenated functionalities within the polymer. These functionalities could be attributed to either oxygenated hydrocarbons or ester groups. The presence of ester groups could further support the notion of PHA units within the polymer backbone, as ester linkages are characteristic features of PHAs. Peaks at m/z 147 and 207 hint at the presence of larger molecular fragments containing multiple oxygen atoms. These fragments suggest the existence of complex structural components within the biopolymer, potentially indicating branching or additional functionalities beyond the basic PHA units. Notably, the m/z 207 peak might correspond to fragments like C6​H7​O​P. This fragment is particularly intriguing, as it potentially points towards the presence of phosphate groups integrated within the biopolymer structure.

The m/z 267 peak points towards fragments containing alkyl chains and potential phosphate groups, such as C8​H11O​P. This suggests the presence of larger sections of the polymer with polyphosphate attachments. Similarly, the m/z 341 peak indicates the presence of complex fragments with both alkyl chains and phosphate groups (C11H15O8​P). These two peaks collectively highlight the extensive phosphorylation throughout the structure of the biopolymer, suggesting a significant degree of phosphate substitution within the PHA segments. The m/z 282 peak likely represents fragments including extended alkyl chains and ester groups. This suggests portions of the polymer backbone with hydroxyl and carboxyl modifications. The presence of these functionalities further supports the notion of PHA units within the polymer structure. The m/z 325 peak strengthens this conclusion, as it potentially corresponds to fragments with significant portions of the polymer backbone, possibly C10H13O8P. This fragment reflects the complex nature of the polymer with multiple ester and phosphate linkages, suggesting a rich tapestry of functional groups within the polymer backbone. Finally, the m/z 429 peak represents a significant and complex fragment, likely including multiple repeating units of the PHA polymer backbone along with polyphosphate groups (C15H25O9P3​). This peak serves as a hallmark feature, highlighting the extensive phosphorylation of the polymer. The sheer size and complexity of this fragment suggest a high degree of phosphate substitution within the PHA segments, potentially influencing the overall properties and functionalities of the biopolymer.

Combining insights from FTIR, ¹H NMR, ¹³C NMR, and GC-MS analyses, the biopolymer can be identified as a complex copolymer with a probable structure of [CH(CH₃)CH₂-O-C(= O)CH₂-CH₂]_n - [PO₃] - [CH(CH₂CH₃)CH₂-O-C(= O)CH₂-CH₂]_m, where n and m represent the chain lengths of the polyhydroxyalkanoate (PHA) segments. This structure suggests the presence of both polyhydroxybutyrate (PHB) and polyhydroxyvalerate (PHV) segments within the polymer backbone, as indicated by CH(CH₃)CH₂ and CH(CH₂CH₃)CH₂, respectively. Additionally, the presence of a phosphate group ([PO₃]) linked to the PHA segments suggests extensive phosphorylation throughout the polymer chain. Notably, the absence of characteristic features such as the amine group (NH₂) indicative of phospholipids in the FTIR spectrum, and the lack of a glycerol backbone signature in both ¹H and ¹³C NMR analyses, definitively excludes the biopolymer from belonging to the phospholipid class.

## Discussion

### Polyhydroxyalkanoate (PHA)-Accumulating Yeast Isolate DMG-2

This study investigates a yeast isolate, DMG-2, with a focus on its ability to accumulate polyhydroxyalkanoates (PHAs). Nile red staining was employed as a primary screening method to identify and confirm the presence of PHAs within microbial colonies. This technique offers several advantages: it is reliable, fast, and reflects the in vivo activity of PHA synthases without inhibiting cell growth^15,16^. Unlike traditional methods dependent on colony opacity or laborious staining procedures, Nile red is incorporated directly into the agar medium, simplifying the detection process. When bound to PHA granules, Nile red emits a strong red fluorescence under UV light, signifying substantial PHA accumulation^15^. This technique provides a rapid and reliable method for screening a large number of isolates for PHA production potential. Furthermore, the in vivo nature of the assay eliminates the need for separate cell extraction steps, offering a more accurate representation of a strain’s PHA biosynthetic capability^17^.

Transmission electron microscopy (TEM) provided valuable insights into the morphology and intracellular localization of PHA granules within DMG-2 cells, complementing the Nile red assay findings. TEM revealed an elongated, oval shape typical of yeast cells, with electron-dense granules likely representing storage materials such as PHAs or polyphosphates. The detailed imagery at the nanometer scale, showing the size, morphology, and intracellular distribution of these granules, supported the identification of PHAs within DMG-2^8,18,19^.

Fourier-transform infrared (FTIR) spectroscopy was used to analyze the chemical composition of the inclusion bodies isolated from DMG-2. The analysis revealed several characteristic peaks indicative of both PHA polymers and polyphosphates. For PHAs, the spectrum displayed prominent absorption peaks at specific wavenumbers, consistent with the presence of functional groups typically found in PHA molecules, including hydroxyl, alkyl, alkene, and carbonyl groups^20,21^. Additionally, peaks associated with P = O and P-O stretching vibrations were observed, suggesting the presence of polyphosphates alongside PHAs^22^. The co-occurrence of polyphosphate inclusions with PHAs suggests a potential interplay between these storage polymers within the yeast cells.

The BLASTN analysis of the 28 S ribosomal RNA sequences from fungal type and reference material revealed *Hanseniaspora valbyensis* NRRL Y-1626 as the predominant match for the DMG-2 sequence, displaying a maximum score of 403 and 99.55% identity. *Hanseniaspora valbyensis* is an unusual yeast species known for its prevalence in traditional balsamic vinegar and cider fermentations. It thrives in environments with high sugar, acid, and pectin contents, where it demonstrates significant pectinolytic activity^23,24^. Additionally, *H. valbyensis* possesses endo-glucanase activity, enabling it to degrade cellulose, and shows tolerance to relatively high levels of selenium^25,26^. Despite not producing high levels of ethanol, *H. valbyensis* synthesizes notable amounts of ethyl and phenethyl acetate, contributing to its unique flavor profile^27^. This yeast’s taxonomic uniqueness is further underscored by its distinctive chromosome composition, with 8 to 9 chromosomes compared to the typical 5 found in other *Hanseniaspora* species^28^.

Consequently, the identification of DMG-2 as the *H. valbyensis* strain expanded our understanding of PHA accumulation capabilities within the Hanseniaspora genus. Notably, the presence of enzymes such as pectinases and endo-glucanases suggested the potential to utilize various carbon sources for PHA production, positioning this strain as a promising candidate for cost-effective bioplastic production from renewable feedstocks. Moreover, the unique chromosomal composition of DMG-2 called for further exploration into its genetic predisposition for PHA biosynthesis. This may have led to the discovery of novel genes or regulatory mechanisms associated with PHA production, as discussed below.

### PHAs biosynthesis genes from yeast strain DMG-2

The study aimed to comprehensively identify and characterize polyhydroxyalkanoate (PHA) biosynthesis genes obtained from the DMG-2 yeast strain, utilizing a diverse range of molecular techniques. Initially, PHA genes were isolated by constructing a genomic library from DMG-2 yeast DNA through partial digestion with *Bam*H1, followed by ligation into the adaptable pBK-CMV vector. This vector, derived from a pUC-based plasmid, exhibits versatility across both eukaryotic and prokaryotic systems, featuring the CMV immediate early promoter for eukaryotic expression and the lac promoter inducible by IPTG for prokaryotic expression^29^. Subsequent screening of colonies using blue/white selection and Nile Red staining assays identified three potential carriers of PHA genes: DMG2-1, DMG2-2, and DMG2-3. Validation of clone DMG2-1, chosen for further analysis, was conducted through transmission electron microscopy (TEM). This analysis demonstrated the efficient biosynthesis and accumulation of PHAs within genetically modified E. coli clone DMG2-1 cells, confirming successful expression of PHA biosynthetic genes from the DMG-2 strain. Notably, the presence of electron-dense granules in the cytoplasm, exhibiting distinct morphology and distribution, provided compelling evidence for the functional integration of these genes.

Verification of selected Escherichia coli clones for yeast contamination, employing one white and one blue clone, was achieved through restriction mapping and 16 S rDNA RFLP analysis. This ensured the purity of the clones and facilitated subsequent molecular investigations^30^. PCR-RFLP analysis targeting 16 S rDNA genes emerged as a pivotal step in confirming the presence of E. coli strains within the yeast-derived genomic library. This method efficiently amplified 1300 bp fragments from both white and blue clones, enabling differentiation of E. coli strains at the genus level through HinfI restriction enzyme digestion. Remarkably, identical RFLP profiles underscored significant genetic similarity among the isolated E. coli clones, highlighting the efficacy of PCR-RFLP analysis in identifying and discerning E. coli strains within complex microbial populations^30^.

The crucial role of the chosen expression vector’s promoter was evidenced by increased RNA band intensity on agarose gels in transgenic clones. Notably, expression levels were significantly higher in M9 minimal media compared to LB media. This observation aligned with findings by Rajacharya et al.^31^, who reported distinct growth patterns and protein expression profiles in E. coli strains under different growth conditions. Their work suggests that minimal media, like M9, may impose a metabolic burden on the cells, prompting prioritization of the expression of genes associated with survival and resource utilization, potentially including the introduced PHA genes.

### Analysis of the ORF1predicted protein sequence

In our investigation, gel electrophoresis was pivotal in identifying a distinct ~ 1.3 kb *Bam*H1 fragment, hypothesized to contain the PHA genes. Subsequent sub-cloning of this fragment into the pUC18 vector enabled thorough DNA sequencing and analysis, offering valuable insights into the genetic composition of our target genes. Through sequence analysis of the DMG-2 insert, following meticulous removal of contaminants and alignment with M13-PCR primers, we revealed a 1035-nucleotide open reading frame named ORF1, encoding a 344-amino acid protein. This discovery implies the presence of a significant protein in our genomic insert, urging us to delve deeper into its functional role and potential impact on cellular metabolism. This entails further sequence analysis at both the protein and genetic levels to decode its function. Further Protein analysis of ORF1 revealed its homology with the PfkB family (residues 23–95) and the ribokinase-like superfamily (residues 7-105), indicating ORF1’s potential role in sugar metabolism through the phosphorylation of sugar molecules. Ribokinases, such as the identified ribulokinase Ydr109c within yeast (Saccharomyces cerevisiae)^32^, highlight a complex interplay between enzyme specificity and cellular needs, suggesting distinct sugar preferences and potential roles in repairing damaged ribulose or participating in unknown metabolic pathways^32,33^. These findings point to a broader family of specialized sugar kinases that may be linked to the synthesis of polyhydroxyalkanoates and polyphosphate metabolism, indicating a multifaceted involvement in cellular metabolic processes.

The PHOBIUS analysis conducted in this study identified transmembrane regions within ORF1, suggesting its probable association with the cell membrane. These regions, spanning residues 119–140, 160–178, and 199–218, delineate ORF1 into cytoplasmic (residues 1-118, 179–198) and non-cytoplasmic (residues 141–159, 219–344) domains. This structural organization implies that ORF1 may participate in sugar metabolism, facilitating interactions both intracellularly and with extracellular molecules. Moreover, considering the relationship between polyhydroxyalkanoate (PHA) synthesis and five-carbon sugar precursors, there are potential links between ORF1 and PHA metabolism. Basnett et al.^34^ categorize PHAs into Short Chain Length (scl-PHAs), comprising three to five carbon atoms per monomer, and Medium Chain Length (mcl-PHAs), consisting of 6 to 14 carbon atoms per monomer. Furthermore, given the observed interplay between polyphosphate metabolism and sugar metabolism across diverse organisms, it is plausible that ORF1’s function extends beyond sugar processing. Integrating insights from Petschnigg et al.^35^, delving into ORF1’s membrane-associated role becomes imperative, as it may yield broader insights into cellular processes, including those involving polyphosphates and PHAs.

Integrated analyses utilizing BLASTAlignPeptide and predicted PHOBIUS transmembrane domains unveil a versatile protein with diverse functions: phosphate hydrolysis, glycosylation, regulatory roles in meiosis and phosphorylation, potential involvement in environmental adaptation, and energy metabolism. These findings suggest a multifaceted role for the protein in various cellular processes, including phosphate metabolism, glycosylation, signaling, environmental adaptation, and potentially polyhydroxyalkanoate (PHA) and polyphosphate synthesis. While ATP isn’t a direct building block for PHAs, it fuels the cellular machinery behind their production, as the metabolic pathways for PHA synthesis require energy, generating precursors and cofactors such as NADPH^36^.

Additionally, the ORF1 protein sequence reveals a putative ATP synthase motif “IVLDWTR” between residues 250–256. This motif deviates from the canonical Walker-B consensus sequence (hhhhDExx) by lacking the aspartate (Asp) at position five^37^. However, the presence of a glutamate (Glu) at position six suggests a potential alternative residue for Mg²⁺ coordination during ATP hydrolysis, aligning with established functionalities of Walker B motifs in various ATPases^38^. The ORF1 sequence extends beyond residue 344, implying additional functionalities beyond Mg²⁺ binding, potentially influencing ATP synthase catalysis and structure. Building upon the identification of a putative Walker B motif in ORF1, PROSITE analysis suggests a layer of complexity through potential post-translational modifications (PTMs).

These include N-glycosylation, phosphorylation by various kinases (cAMP-dependent protein kinase, protein kinase C, casein kinase II), N-myristoylation, and amidation. Such PTMs, could significantly influence protein stability, localization, and interaction with other molecules. This interplay between a putative ATPase domain and potential PTMs underscores the potential for intricate regulation of ORF1 function within the cell^39,40^. Interestingly, PSI-BLAST alignment and the presence of a Walker B motif point towards ORF1’s role in ATP metabolism, similar to known kinases like adenosine kinase and ribokinase. This suggests ORF1 might not only be an ATP-binding protein but also possess enzymatic activity for ATP hydrolysis. This found knowledge of ORF1’s dual functionality, potentially interacting with protein kinases through its flexible regions while playing a role in cellular energy through ATP metabolism, highlights its significant role in cellular processes^41^.

The Swiss-modeling analysis of ORF1, utilizing the Escherichia coli phosphofructokinase-2 template (3uqd.1.D), reveals significant insights into its structure and function. Notably, residues 23–95 exhibit high structural similarity to the template, suggesting ORF1’s involvement in sugar metabolism and ATP hydrolysis. This inference is bolstered by the identification of the ATP synthase motif “IVLDWTR” within residues 250–256, indicating a critical role in ATP binding and hydrolysis. The presence of magnesium ions in the template further underscores ORF1’s enzymatic activity and stability. The absence of predicted models for other segments of ORF1 may result from post-translational modifications such as phosphorylation and glycosylation. These modifications suggest a dynamic structural nature, highlighting ORF1’s involvement in various cellular energy metabolism and regulatory processes. The potential for ATP binding and hydrolysis, along with its influence on sugar metabolism, implies ORF1’s significant roles in polyhydroxyalkanoate (PHA) and polyphosphate biosynthesis pathways. This interpretation aligns with the findings of Chen et al.^42^, who studied the impact of MOF-808 (a metal-organic framework) on PHA production. Their research demonstrated that MOF-808 enhances ATP supply, regulates coenzyme A metabolism, and modulates PHA synthesis pathways. This supports the hypothesis that ORF1 plays a crucial role in PHA biosynthesis by ensuring an adequate ATP supply, thereby regulating the metabolic pathways involved in PHA production.

### Genomic analysis of the ORF1 DNA sequence

The genomic decoding of ORF1 unveils its multifaceted involvement in mRNA processing, metabolic regulation, and epigenetic control. Identified polyadenylation and splice sites indicate ORF1’s crucial roles in mRNA stability and splicing, essential for proper gene expression. Moreover, ORF1’s participation in metabolic regulation, particularly in PHA synthesis pathways, is underscored by the presence of transcription factor binding site motifs, such as those for CREB and ChREBP, emphasizing its importance in managing carbon flux for sustainable bioplastic manufacturing. Additionally, the presence of CpG islands within ORF1 suggests potential epigenetic regulation, potentially through DNA methylation-mediated gene expression modulation^43^. Protein analysis further reveals ORF1’s involvement in sugar metabolism and energy production through homology to carbohydrate kinase, ribokinase-like domains, and structural similarities to phosphofructokinase-2. These combined insights position ORF1 as a pivotal regulator bridging metabolic and epigenetic landscapes, influencing pathways like PHA biosynthesis and highlighting its multifunctional cellular roles.

The in silico analysis of the ORF1 promoter region reveals several key elements characteristic of eukaryotic promoters, including the presence of putative TATA and BRE boxes, as well as the core promoter element Inr and a putative DPE sequence^44^. Notably, a motif within the promoter bears resemblance to Upstream Activating Sequences (UAS, UASALK) elements found in yeast, particularly the Overlapping Replication Element (ORE) in Saccharomyces cerevisiae, which typically features inverted CGG triplets separated by 15–18 nucleotides^14^. This motif suggests potential regulatory similarities between ORF1 transcriptional regulation and yeast transcriptional regulation mediated by UAS elements. Furthermore, the discovery of UAS ALK-dependent transcriptional activation by short-chain fatty acids like butyrate and propionate offers insights into their potential role in polyhydroxyalkanoates (PHA) and polyphosphate synthesis pathways.

As these carbon sources induce peroxisome proliferation in Candida tropicalis, their utilization initiates fatty acid β-oxidation within peroxisomes, leading to the generation of butyryl- or propionyl-CoA^14^. These metabolic intermediates could serve as precursors for PHA biosynthesis, contributing to the production of biopolymers. Additionally, the activation of transcriptional machinery through UASALK suggests a link between carbon source utilization and the regulation of genes involved in polyphosphate metabolism, potentially influencing cellular energy storage and metabolic regulation. Thus, the observed transcriptional response to short-chain fatty acids underscores their significance in orchestrating cellular processes relevant to biopolymer synthesis and cellular metabolism.

The computational analysis of the promoter region of the Open Reading Frame 1 (ORF1) has provided significant insights into its regulatory architecture. Key elements typical of eukaryotic promoters have been identified, including the TATA box, BRE box, Initiator (Inr) element, and a putative Downstream Promoter Element (DPE) sequence. These elements are crucial for the accurate positioning of RNA polymerase II and the initiation of transcription^44^. Furthermore, the analysis revealed a specific motif within the ORF1 promoter region that resembles the Upstream Activating Sequences (UAS) found in yeast. This motif, characterized by inverted CGG triplets separated by 15–18 nucleotides, is similar to the Overlapping Replication Element (ORE) in Saccharomyces cerevisiae^14^. The presence of this motif suggests functional parallels between the transcriptional regulation of ORF1 and the mechanisms mediated by UAS elements in yeast. Interestingly, UAS ALK-dependent transcriptional activation can be triggered by short-chain fatty acids, such as butyrate and propionate. These fatty acids are known to induce peroxisome proliferation in Candida tropicalis, initiating fatty acid β-oxidation within these organelles. This process produces butyryl-CoA or propionyl-CoA, which are metabolic intermediates that can serve as precursors for polyhydroxyalkanoate (PHA) biosynthesis, thus contributing to biopolymer production^14^.

Moreover, a motif AGAAAG discovered through MEME analysis potentially binds to the transcription factor PRDM1, a C_2_H_2_-type zinc finger transcription factor, as indicated by the Tomtom motif comparison. This suggests a role in regulating genes associated with PHA synthesis and polyphosphate metabolism, which are crucial for various cellular processes and biopolymer production^45^. The research by Chen et al.^45^ on the C_2_H_2_-type zinc finger transcription factor Fts2 in Yarrowia lipolytica revealed its function as a transcriptional repressor that influences filamentation and expression pathways. This underscores the conservation of regulatory mechanisms across species and highlights the importance of zinc finger transcription factors in fungal gene regulation. The presence of zinc finger transcription factors and their cognate DNA-binding motifs, such as the C6 zinc cluster motif^45^ acting as a UAS activator for ORF1, emphasizes their critical role in coordinating transcriptional responses to environmental cues, metabolic signals, and developmental processes. Their interactions with regulatory elements like promoters and enhancers are essential for fine-tuning gene expression programs, which are vital for cellular homeostasis and adaptation.

Comparison of the FTIR spectra between transgenic *E. coli* and wild-type yeast DMG-2 reveals both similarities and distinctions in their molecular compositions and structural features. Both spectra exhibit peaks indicative of common functional groups, such as hydrogen-bonded O-H and C-H stretching vibrations, suggesting the presence of hydroxyl and alkyl groups in the respective biopolymers, PHA, and polyphosphate. Additionally, shared peaks related to C = O and P = O stretching vibrations denote the presence of carbonyl and phosphate groups in both organisms^20–22^. However, distinct peaks in transgenic *E. coli*, including those corresponding to C = C and C-H bending vibrations, suggest structural variances compared to wild-type yeast. Conversely, additional peaks observed in the wild-type yeast spectrum, particularly at higher wavenumbers, may indicate unique molecular species or structural components absent in *E. coli*. Moreover, the prevalence of more phosphate-related peaks in the wild-type yeast spectrum implies a potentially more complex molecular composition of polyphosphate in yeast compared to *E. coli*. This detailed comparison provides insights into the effects of genetic modification on biopolymer production and highlights the intricacies of cellular metabolism and molecular architecture in microbial systems.

The comprehensive analysis of the 1 H and 13 C NMR spectra of the transgenic *E. coli* co-polymer unveils the structural diversity of polyhydroxyalkanoates (PHAs) within the polymer matrix. Distinct peaks in the 1 H NMR spectrum, such as those corresponding to terminal methyl groups (-CH₃), methylene groups (-CH₂-), and methine protons (-CH-), signify the presence of various PHA types. Similarly, the 13 C NMR spectrum delineates unique chemical environments associated with different PHA segments, including terminal methyl carbons, repeating methylene units in the polymer backbone, and methylene carbons adjacent to ester linkages.

A complex biopolymer structurally identified as a copolymer of polyhydroxybutyrate (PHB) and polyhydroxyvalerate (PHV) segments was produced by transgenic Escherichia coli. Extensive phosphate groups were found to be attached to the polymer backbone, confirmed by Fourier-transform infrared spectroscopy (FTIR) identifying hydroxyl, alkyl, carbonyl, and phosphate groups, and further supported by Gas chromatography-mass spectrometry (GC-MS) revealing hydroxyalkanoate units, oxygenated functionalities, and phosphate groups^46–50^. Nuclear magnetic resonance (NMR) analysis (implied but not explicitly mentioned) likely corroborated the structure by revealing terminal methyl, methylene, methine units, and ester linkages. The complete structure of the biopolymer can be represented as: [CH(CH₃)CH₂-O-C(= O)CH₂-CH₂]_n - [PO₃] - [CH(CH₂CH₃)CH₂-O-C(= O)CH₂-CH₂]_m. This highly phosphorylated PHA copolymer exhibits unique properties due to the interplay between its structural elements. The extensive phosphate groups, as evidenced by various spectroscopic techniques, contribute to its high hydrophilicity, hindering tight packing of polymer chains and preventing crystallization^51–54^. This combined with the inherent structural irregularities of the amorphous PHB-PHV copolymer, results in a flexible material resistant to solidification into a powder upon solvent evaporation. Notably, the absence of the amine group (NH₂) characteristic of phospholipids in the FTIR spectrum and the lack of a glycerol backbone signature in both ¹H and ¹³C NMR analyses definitively exclude the biopolymer from this class.

In general, the biopolymer’s complex structure, characterized by the presence of both PHB and PHV segments alongside extensive phosphate modifications, suggests potential applications in biotechnology and industry where tailored material properties are desired. Hydrophilic polyhydroxyalkanoates (PHAs) have diverse applications due to their biocompatibility, biodegradability, and hydrophilic properties. In the biomedical field, they are utilized for developing drug delivery systems, tissue engineering scaffolds, and wound dressings, offering controlled release and promoting tissue regeneration^55^. In environmental technology, PHAs serve as sustainable alternatives to conventional plastics, contributing to waste reduction and pollution control^56^. Additionally, their use in food packaging helps extend shelf life and ensures food safety^57^. These versatile applications highlight the potential of hydrophilic PHAs in advancing both medical innovations and environmental sustainability.

### Potential role of ORF1 in PHA biosynthesis

The identified ORF1 encodes a protein with similarities to carbohydrate kinase and ribokinase-like domains, indicating its involvement in sugar metabolism. This protein is likely responsible for converting carbon sources into intermediates that could serve as precursors for polyhydroxyalkanoate (PHA) synthesis. Specifically, these domains imply that ORF1 might phosphorylate sugars, producing crucial intermediates such as acetyl-CoA and propionyl-CoA. These molecules are essential for various metabolic pathways, including PHA synthesis, as acetyl-CoA is a central precursor in producing fatty acids and other biomolecules^62,63^. Moreover, CoA esters like acetyl-CoA are vital for metabolic processes such as the citric acid cycle and fatty acid biosynthesis. The synthesis of these CoA intermediates is key for energy production and generating metabolic precursors necessary for biopolymer biosynthesis, including PHAs^64^.

PHAs are microbial storage polymers synthesized under nutrient-limited conditions, typically by PHA synthase enzymes. However, recent evidence suggests that alternative pathways for PHA polymerization might exist, particularly in systems lacking conventional PHA synthases. The transmembrane nature of the ORF1-encoded protein suggests it may interact with other membrane-associated proteins, potentially facilitating PHA monomer polymerization through an alternative enzymatic or protein complex pathway^65,66^.

Genomic analysis of ORF1 has identified several transcription factor binding sites (TFBS) and regulatory motifs linked to carbon metabolism. These elements suggest that ORF1 is part of a broader regulatory network governing carbon flux, which is crucial for providing PHA precursors like acetyl-CoA. The presence of TFBS motifs in organisms such as maize and Arabidopsis —known for their roles in carbohydrate metabolism—implies that ORF1’s expression might be connected to cellular energy demands^67,68^. Additionally, the identification of CpG islands within the ORF1 sequence supports its involvement in gene regulation. CpG islands are known to control gene transcription through epigenetic mechanisms like DNA methylation. The regulatory influence of these CpG islands may modulate ORF1’s transcriptional activity, affecting the production of PHA precursors and potentially influencing PHA polymerization via alternative pathways^69^.

## Conclusion

This study underscores the potential of the yeast isolate DMG-2, identified as *Hanseniaspora valbyensis*, for sustainable bioplastic production through the accumulation of polyhydroxyalkanoates (PHAs). Screening methods, including Nile red staining, TEM, and FTIR analyses, confirmed the presence of electron-dense PHA granules and polyphosphates within the cells, indicating a complex relationship between these storage polymers. Genomic analysis of the DMG-2 strain, focusing on the ORF1 gene and its predicted protein sequence, revealed its crucial role in sugar metabolism and ATP hydrolysis, which are essential for PHA synthesis. The potential gene’s involvement in both PHA biosynthesis and polyphosphate metabolism highlights its significance in microbial bioplastic production. The biopolymer produced by wild-type DMG-2 and genetically modified *E. coli* expressing its PHA biosynthetic genes was characterized by enhanced hydrophilicity and flexibility, making it suitable for various industrial and biomedical applications. The study also explored the PHA biosynthesis pathway in *H. valbyensis* DMG-2, revealing differences from the well-known bacterial mechanisms. Unlike bacteria, which typically use PHA synthase to convert acetyl-CoA and propionyl-CoA into PHAs, *H. valbyensis* DMG-2 appears to utilize a distinct metabolic network. The ORF1 gene, homologous to enzymes involved in sugar metabolism, suggests an alternative pathway incorporating phosphate groups into PHA precursors, resulting in phosphorylated PHA derivatives. This highlights the potential role of phosphate metabolism in this unique biosynthetic process. The specialized metabolic environment of *H. valbyensis*, thriving in sugar- and acid-rich niches, may contribute to its distinctive PHA production. This discovery paves the way for new metabolic engineering strategies, enhancing our understanding of PHA biosynthesis across different organisms. By leveraging the unique pathway in *H. valbyensis* DMG-2, the scientific community can explore innovative methods for producing sustainable, environmentally friendly bioplastics with tailored properties.

## Materials and methods

### Screening for PHA-producing wild-type yeast

#### Nile red staining assay

Over 100 yeast isolates from the lab culture collection were screened for PHA production using a Nile Red staining assay incorporated into the growth medium. YPD agar medium was prepared by dissolving 20 g/L peptone, 20 g/L glucose, and 10 g/L yeast extract in deionized water. The solution was then heated, adjusted to pH 5.6, and supplemented with 20 g/L agar before autoclaving for sterilization. After cooling, a Nile Red working solution (0.25 mg/mL in sterile DMSO) was added to the sterilized YPD agar to achieve a final concentration of 0.5 µg dye/mL medium^15^. The Nile Red-amended YPD agar was then poured into Petri dishes and allowed to solidify. Individual yeast isolates were inoculated onto separate plates and incubated for 24–48 h at 30 °C. After incubation, plates were exposed to UV light (312 nm) to visualize red fluorescence within the colonies, indicating potential PHA accumulation^6^. This combined approach of Nile Red staining within the YPD agar medium facilitated rapid screening of a large number of yeast isolates for potential PHA production, allowing for further confirmation through alternative methods (e.g. Sudan Blue and Sudan Black B).

### Identification of positive isolate DMG-2

Among the yeast isolates screened using the Nile Red assay, the isolate exhibiting the brightest orange fluorescence under UV light, designated DMG-2, was selected for further identification. To achieve this, PCR amplification of the 28 S rRNA gene was performed using the intergenic spacer region (ITS1/ITS4 primers) as described by Zarrin et al.^58^. A 25 µL reaction mixture containing reaction buffer, dNTPs, MgCl2, Taq polymerase, DMG-2 genomic DNA, and nuclease-free water was subjected to thermal cycling (MJ Research, model PTC-200 Peltier, USA) with the following program: initial denaturation (95 °C, 3 min), followed by 35 cycles of denaturation (95 °C, 1 min), annealing (50 °C, 30 s), extension (72 °C, 2 min), and a final extension (72 °C, 10 min). The resulting PCR products were visualized on a 0.8% agarose gel and subsequently sequenced using an automated DNA sequencer (3130 genetic analyzer, Japan). The obtained 28 S rDNA sequence was submitted to the National Center for Biotechnology Information (NCBI) for identification and distance tree construction using the neighbor-joining method.

### Transmission Electron Microscopy (TEM) analysis

To definitively confirm the presence and intracellular distribution of PHAs within DMG-2 cells, TEM analysis was employed. Harvested cells were pre-fixed with 2.5% glutaraldehyde in 0.1 M sodium cacodylate buffer (pH 7.4) for 2 h at 4 °C to preserve morphology. Subsequent fixation with 1% osmium tetroxide (OsO_4_) in 0.1 M sodium cacodylate buffer (pH 7.4) for 2 h at 4 °C further enhanced contrast, particularly for membranes and PHA inclusions. Dehydration was achieved through a graded ethanol series (30%, 50%, 70%, 90%, and 100% ethanol) for 10 min each, followed by infiltration with Spurr’s resin (mixed with fresh resin at a 1:1 ratio) for 1 h and pure Spurr’s resin overnight. Ultrathin Sect. (70 nm) were obtained using a microtome and mounted on copper grids for stability and electron conductivity. Finally, double-staining was performed with 2% uranyl acetate in 50% ethanol for 15 min and lead citrate (saturated solution) for 5 min to enhance the contrast of cellular components, allowing visualization of PHAs under the TEM at an accelerating voltage of 120 kV.

### PHA production and FTIR analysis

DMG-2, the yeast isolate exhibiting the strongest PHA production based on the Nile Red assay, was subjected for PHA FTIR spectroscopic analysis. A seed culture of DMG-2 was first grown in 50 mL YPD medium (20 g/L glucose, 20 g/L peptone, 10 g/L yeast extract) at 30 °C and 150 rpm for 24 h in a shaking incubator. This seed culture was then used to inoculate 450 mL of sterilized M9 fermentation medium supplemented with 4 g/L glucose (12.8 g/L Na_2_HPO_4_, 3.1 g/L KH_2_PO_4_, 0.5 g/L NaCl, 1 g/L NH_4_Cl, and 0.5 g/L MgSO_4_·7H_2_O). The inoculated cultures were incubated in 2000 mL Erlenmeyer flasks on a shaking incubator at 30 °C and 150 rpm for 72 h. Following fermentation, cells were harvested by centrifugation (Model: 3–16 K, Sigma, Germany) at 5,000 xg for 10 min to pellet the biomass.

The cell pellet was then washed with sterile distilled water to remove residual medium components. After 3 times washes, the cells were re-suspended and dried at a low temperature 40 °C (Oven (Heraeus Instrument, Germany)) for overnight to remove water content and facilitate complete cell wall disruption for subsequent PHA extraction. Cell disruption for PHA extraction was achieved by sonication using an Ultrasonicator (HD220 Max power BANDELIN SONOPULS, Berlin, Germany) in a 50 mL chloroform solution for 10 min. The cell suspension was then incubated for 24 h at 30 °C to facilitate complete PHA extraction. Finally, cell debris was removed by centrifugation, and the purified PHA was obtained from the clear supernatant after filtration the purified PHA was obtained from the clear supernatant after filtration through a Whatman No. 3 filter (International Lt, England) and solvent evaporation at room temperature. The refined polyhydroxyalkanoate (PHA) was meticulously preserved at 4 °C to maintain its structural integrity for subsequent spectroscopic analysis. Notably, an intriguing observation emerged during this process: contrary to initial expectations, the polymer did not transition into a powdered form following the evaporation of chloroform. This unexpected phenomenon warrants comprehensive exploration and discussion in the subsequent section, shedding light on the distinctive characteristics of this particular PHA variant.

For Fourier-Transform Infrared (FTIR) analysis, a Bruker IR spectrometer was employed to identify functional groups within the purified PHA. Here, 2–5 mg of the purified PHA sample was dissolved following the method described by Ravenelle and Marchessault^59^ in 1 mL of chloroform (CHCl_3_) to create a 2–5% (w/v) solution. A small aliquot (2–3 drops) of this solution was then carefully deposited onto a clean and dry sodium chloride (NaCl) disk. The solvent was allowed to evaporate completely under ambient conditions or in a desiccator to ensure complete removal of residual moisture. The NaCl disk with the deposited PHA film was then placed in the sample compartment of the FTIR spectrometer purged with nitrogen gas. An infrared spectrum was collected over a wavenumber range of 4000 –400 cm-1 at a high resolution of 4 cm-1.

### Isolation and characterization of putative PHA biosynthesis genes

#### Construction of a genomic library and screening for PHA genes

A genomic library was constructed by partially digesting DMG-2 yeast genomic DNA with *Bam*H1 restriction enzyme (10 U/µg DNA) for 1 h at 37 °C. The digested fragments were purified using a commercially available DNA purification kit (Gene JET™ PCR gel purification kit) following the manufacturer’s instructions. The purified fragments were then ligated at high concentration (100 ng insert DNA and 1 µg vector DNA) into the *Bam*H1-linearized cloning vector, pBK-CMV (Stratagene, La Jolla, CA, USA). This vector contains a lacZα gene fragment that allows for blue-white color selection. During the ligation reaction, insertion of genomic DNA fragments disrupts the lacZα gene, preventing the production of β-galactosidase and resulting in white colonies. In contrast, colonies containing the vector without an insert will express the lacZα gene and produce blue colonies. The ligation reaction was carried out using T4 DNA ligase (1–2 Weiss units/µL) and incubated overnight at 16 °C. The ligation products were then transformed into competent cells and plated on LB agar plates (containing g/l; 10 g/l peptone, 10 g/l NaCl, 5 g/l yeast extract, and 20 g/l (w/v) agar) supplemented with 50 µg/mL X-gal (5-bromo-4-chloro-3-indolyl-β-D-galactopyranoside) and 0.1 mM IPTG (isopropyl β-D-1-thiogalactopyranoside) for blue-white color selection of recombinant colonies^60^.

Competent *E. coli* JM107 cells (Sigma) were prepared using the calcium chloride method^60^ and transformed with the ligation mixture containing the putative PHA genes. Briefly, an aliquot 50 µL of competent cells was mixed with the ligation mixture (containing purified DNA fragments and *Bam*H1-linearized pBK-CMV vector in standard concentration, typically 100 ng insert DNA and 1 µg vector DNA) and incubated on ice for 30 min. A heat shock was then applied at 42 °C for 45 s to facilitate plasmid DNA uptake. The cells were immediately placed back on ice for 2 min before being plated onto LB agar plates (g/l; 10 g/l peptone, 10 g/l NaCl, 5 g/l yeast extractand 20 g/l (w/v) agar was added to the above components before autoclaving) containing the selection marker (50 µg/mL kanamycin for pBK-CMV). After transformation, white colonies were screened for the presence of PHA genes using Nile Red staining^15^. Briefly, selected colonies were grown overnight in LB broth (containing g/L; 10 g/L peptone, 10 g/L NaCl, 5 g/L yeast extract), harvested by centrifugation at 5,000 x g for 10 min, and stained with a Nile Red solution (1 µg/mL in acetone) for 15 min. Colonies exhibiting orange fluorescence under UV light were considered positive for PHA production and selected for further analysis.

#### Validation and subcloning of positive clones

Selected colonies were validated through restriction mapping analysis. Plasmid DNA was isolated from positive clones using a commercially available plasmid miniprep kit (QIAprep Spin Miniprep Kit, Qiagen) following the manufacturer’s instructions. Restriction mapping involved digesting the isolated plasmid DNA with restriction enzyme *Bam*H1 under recommended buffer and incubation conditions defined by the enzyme supplier (Stratgen). The digested DNA fragments were then separated using gel electrophoresis on 1% agarose gels stained with ethidium bromide (10 µg/mL) and visualized under UV light. The presence and size of the inserted DNA fragment within the plasmid confirmed the presence of potential PHA genes.

To definitively confirm successful *E. coli* transformation with the isolated DNA fragment and differentiate it from the original DMG-2 yeast, a two-step approach was employed. First, PCR amplification of the 16 S ribosomal RNA gene using universal eubacterial primers^30^ a ~ 1.3 kb fragment specific for bacteria, ensuring no amplification from the eukaryotic DMG-2 yeast. Subsequent restriction fragment length polymorphism (RFLP) analysis of the purified PCR product involved digestion with HinfI (2 U) under recommended conditions (Stratagene). This enzyme cleaves conserved sequences within the *E. coli* 16 S rRNA gene, generating a characteristic restriction profile with fragment sizes consistent across the *E. coli* genus^30^. The digested fragments were then separated on a 2% agarose gel containing ethidium bromide for visualization. A positive PCR product and a distinct *Hin*fI profile compared to the undigested PCR product would confirm the presence and *E. coli* origin of the transformed DNA fragment, complementing the blue-white colony selection method. This combined approach definitively confirms *E. coli* transformation with the desired fragment, likely from a bacterial source distinct from DMG-2 yeast.

Following validation, a single clone harboring a distinct ~ 1.3 kb *Bam*H1 fragment, designated DMG2-1 and putatively containing the PHA genes, was selected for further analysis. This fragment was gel-purified, while a PUC18 vector (Promega, USA) was linearized with *Bam*HI and dephosphorylated. The purified insert DNA was then ligated with the linearized and dephosphorylated PUC18 vector at a 1:3 molar ratio (insert: vector) overnight at 14°C using T4 DNA ligase. Competent *E. coli* cells were transformed with the ligation mixture and selected on LB agar containing ampicillin. Positive clones were identified by white/blue assay and used to isolate plasmid DNA. Finally, the insert within the plasmid was amplified using universal M13 primers (M13-Forward: 5’-GTAAAACGACGGCCAGT-3’ and M13-Reverse: 5’-CAGGAAACATCGGTAACC-3’) flanking the cloning sites in the PUC18 vector. The PCR products were purified and sequenced using an automated DNA sequencer (3130 Genetic Analyzer).

### Expression analysis and functional validation of putative PHA genes

To investigate the effectiveness of the pBK-CMV promoter in driving expression of the putative PHA genes and correlate it with RNA abundance on agarose gels, a single, brightly red fluorescent *E. coli* DMG2-1 clone harboring the pBK-CMV vector with the inserted DNA fragment was selected. This clone was cultured in two different media: LB broth and M9 minimal media. The rationale behind using these media is that LB broth is a rich media providing all essential nutrients for bacterial growth, while M9 minimal media is a defined media with limited nutrients, potentially inducing the expression of genes on the plasmid for survival. Following controlled cultivation under appropriate growth conditions (37 °C with shaking), plasmid DNA was extracted from the cultures grown in each media type using a commercially miniprep kit following the manufacturer’s instructions (described above). The extracted DNA was then analyzed using gel electrophoresis on a 0.1% agarose gel stained with ethidium bromide (typically 0.5 µg/mL) for visualization under UV light. This analysis assessed the presence and size of the plasmid DNA in each sample, aiming to correlate the integrity of the plasmid with the media type. If the pBK-CMV promoter is functional and responds to nutrient limitations, we expect to see intact plasmid DNA in both LB and M9 cultures. However, a stronger expression of the putative PHA genes in M9 minimal media might be reflected by a higher abundance of the corresponding RNA species visualized on agarose gel.

#### Confirmation of PHA production using TEM

To definitively confirm intracellular PHA production in the transgenic *E. coli* DMG2-1 clone, transmission electron microscopy (TEM) analysis was employed, following the established protocol utilized for the wild-type yeast isolate DMG-2. The DMG2-1 clone was cultured in M9 minimal media supplemented with glucose as the carbon source. The use of minimal media with a single carbon source is a key nutritional limitation known to induce PHA accumulation in *E. coli*. After growth, the cells were harvested, fixed with glutaraldehyde, dehydrated through graded ethanol washes, and infiltrated with Spurr’s resin for sectioning. Ultrathin sections were then stained with heavy metals and visualized under TEM. The presence and morphology of electron-lucent inclusions within DMG2-1, consistent with observations from the wild-type yeast, would confirm successful expression and functionality of the cloned PHA genes in the engineered *E. coli* strain.

#### Analysis of DNA and its predicted protein sequences of ORF1

A comprehensive bioinformatics analysis was conducted on the insert DNA sequence derived from the yeast isolate DMG-2. This sequence was expressed in the *E. coli* transgenic clone DMG2-1 and subcloned in pUC-18 plasmid, followed by sequencing using M13 primers, resulting in a sequence of approximately 1379 base pairs. Various specialized tools were employed for this analysis. Checking for vector contamination was performed using NCBI VecScreen program (https://www.ncbi.nlm.nih.gov/tools/vecscreen/). The NCBI ORF Finder (https://www.ncbi.nlm.nih.gov/orffinder) identified open reading frames within the DNA sequence (ORF1 = 1035 bp, submitted GenBank accession number PP869688), leveraging the standard genetic code to pinpoint start and stop codons. InterPro (https://www.ebi.ac.uk/interpro/) was employed to predict protein functional domains of ORF1 by integrating multiple protein signature databases, providing insights into potential protein functions and family classifications. PHOBIUS (https://phobius.sbc.su.se/) predicted transmembrane regions and signal peptides, crucial for understanding protein localization and topology. PROFsec (https://rostlab.org/services/prof/) was utilized to predict detailed structural elements of the protein. The multifunctional protein was identified through a combination of bioinformatics analyses, utilizing the BLASTAlignPeptide tool (https://www.uniprot.org/blast) to align the protein sequence against the UniProt database. PROSITE (https://prosite.expasy.org/) identified specific protein motifs and potential post-translational modification sites essential for protein function and regulation. Swiss-Model (https://swissmodel.expasy.org/) provided structural modeling based on homologous protein templates, offering three-dimensional insights into protein structure and potential functional sites.

To decode the genomic sequence, multiple bioinformatics tools from Softberry (http://softberry.com/) were employed. The POLYAH tool identified potential polyadenylation sites, predicting regions for cleavage and polyadenylate tail addition to mRNA transcripts. FSPLICE 1.0 analyzed splice sites in genomic sequences from Schizosaccharomyces pombe and Saccharomyces cerevisiae, identifying critical donor and acceptor sites for mRNA processing. Nsite Version 6.2014 revealed transcription factor binding sites (TFBS), highlighting regulatory elements that might influence metabolic pathways, including polyhydroxyalkanoates (PHA) synthesis. CpGFinder detected CpG islands, indicating potential regulatory regions within the DNA sequence. Neural Network Promoter Prediction (NNPP) (http://www.fruitfly.org/seq_tools/promoter.html) identified a putative promoter region, revealing key elements like the TATA box, BRE motif, and initiator sequence essential for transcription initiation. Finally, MEME analysis (http://meme-suite.org/tools/meme) identified a conserved nucleotide motif, and Tomtom software (http://meme-suite.org/tools/tomtom) revealed a significant match with a known transcription factor binding motif.

#### Spectroscopic Analysis of Polyhydroxyalkanoates (PHAs)

The transgenic *E. coli* clone DGM2-1, containing the PHA genes from DGM-2, was cultivated to produce PHA. A 50 mL LB seed culture was utilized to inoculate 450 mL of sterilized M9 fermentation media, enriched with glucose as the carbon source. Both LB and M9 media were supplemented with kanamycin (50 µg/mL). After 72 h of shaking incubation at 30 °C and 150 rpm, cells were harvested, washed, and dried. Cell wall disruption was achieved by sonication, followed by 24-hour incubation at 30 °C for PHA extraction. Cell debris was removed, and purified PHA was obtained from the clear supernatant after filtration and chloroform evaporation at room temperature. The resulting PHA, resembling the form typically produced from the wild type strain DMG-2 rather than solidifying into a powder, was stored at 4 °C for spectroscopic analysis.

The infrared (FTIR) spectroscopic analysis was conducted as described above with the wild-type yeast isolate DMG-2. For this analysis, PHA samples from clone DGM2-1 were dissolved in chloroform and deposited as a film on a sodium chloride disk following the method described by Ravenelle and Marchessault^59^. The NMR spectra were recorded on a JOEL ECA 500 spectrometer. For ^1^H-NMR spectroscopy, a 500 MHz frequency was utilized. The spectra were obtained from a CDCl3 solution of PHA at a concentration of 30 mg/ml at 20 °C, with an acquisition time of 1.30809856 s and a spectral width of 12.5250501 kHz. For ^13^C-NMR spectroscopy, the spectra were also recorded from a CDCl_3_ solution using ^1^H-decoupling, with an acquisition time of 0.69206016 s and a spectral width of 47.348485 kHz. Chemical shifts for ^1H-NMR and ^13^C-NMR were referenced to chloroform (CHCl_3_) and deuterated chloroform (CDCl_3_) at 7.24 ppm and 77.0 ppm, respectively. These methods were performed following standard protocols for FTIR and NMR spectroscopic analyses.

PHA samples from clone DGM2-1 were qualitatively and quantitatively analyzed using Gas Chromatography-Mass Spectrometry (GC-MS). Liquid cultures were centrifuged at 10,000 g for 15 min. The resulting cell pellets were washed twice with saline solution and then lyophilized overnight. Approximately 8–10 mg of the lyophilized cell material was subjected to methanolysis in the presence of 15% (v/v) sulfuric acid, converting the constituent 3-hydroxyalkanoic acids to their methyl esters. This process followed the method described by Qi and Rehm^61^. For GC analysis, 3 µl of the sample was injected into a Hewlett-Packard 6890 gas chromatograph-mass spectrometer (Hewlett-Packard, Palo Alto, CA, USA). The internal standard used for quantification was methyl heptadecanoate, while the external standard consisted of a mixture of known PHA monomers (sigma). The analysis was conducted using the same column and temperature profile as for the GC analysis. Derivatizations of the methyl esters of monomer constituents with pentamethylsilyl (PMSi).

## Data Availability

Data Availability Statement: All data generated or analyzed during this study are comprehensively documented within the manuscript. Moreover, the DNA sequences showcased in this research have been diligently deposited in GenBank with the accession numbers AC PP869688 for ORF1 and AC PP865075 for the yeast strain DMG-2. These resources are readily accessible for additional scrutiny and validation by the scientific community.
